# Three-phase flow displacement dynamics and Haines jumps in a hydrophobic porous medium

**DOI:** 10.1098/rspa.2020.0671

**Published:** 2020-12-23

**Authors:** Abdulla Alhosani, Alessio Scanziani, Qingyang Lin, Ahmed Selem, Ziqing Pan, Martin J. Blunt, Branko Bijeljic

**Affiliations:** 1 Department of Earth Science and Engineering, Imperial College London, London, UK; 2 Department of Chemical Engineering, Imperial College London, London, UK; 3 State Environmental Protection Engineering Center for Coal-Fired Air Pollution Control, Zhejiang University, Hangzhou 310027, People's Republic of China

**Keywords:** three-phase flow, synchrotron imaging, enhanced oil recovery, gas injection, porous media, wettability

## Abstract

We use synchrotron X-ray micro-tomography to investigate the displacement dynamics during three-phase—oil, water and gas—flow in a hydrophobic porous medium. We observe a distinct gas invasion pattern, where gas progresses through the pore space in the form of disconnected clusters mediated by double and multiple displacement events. Gas advances in a process we name three-phase Haines jumps, during which gas re-arranges its configuration in the pore space, retracting from some regions to enable the rapid filling of multiple pores. The gas retraction leads to a permanent disconnection of gas ganglia, which do not reconnect as gas injection proceeds. We observe, *in situ*, the direct displacement of oil and water by gas as well as gas–oil–water double displacement. The use of local *in situ* measurements and an energy balance approach to determine fluid–fluid contact angles alongside the quantification of capillary pressures and pore occupancy indicate that the wettability order is oil–gas–water from most to least wetting. Furthermore, quantifying the evolution of Minkowski functionals implied well-connected oil and water, while the gas connectivity decreased as gas was broken up into discrete clusters during injection. This work can be used to design CO_2_ storage, improved oil recovery and microfluidic devices.

## 1. Introduction

Understanding the pore-scale physics governing the simultaneous flow of three fluid phases—two immiscible liquids and a gas—inside porous structures is of great interest to many areas of science and technology, including microfluidic devices [1], packed bed chemical reactors [2] and catalysis [3]. In addition, the dynamics of multiphase flow at the micro-scale (pore scale) regulate the movement of fluids in large, extensive natural porous systems, for instance during carbon dioxide storage in geological reservoirs to prevent dangerous global warming [4–8], and gas injection (GI) in oilfields as an enhanced oil recovery (EOR) method [9,10], as well as the removal of non-aqueous phase liquid (NAPL) in soils [11].

The main objective of our work is to use fast synchrotron X-ray micro-tomography to characterize the key physical processes that control the pore-scale dynamics during three-phase flow in hydrophobic porous media, namely wettability order, spreading layers and double/multiple displacement events [12]. For simplicity, throughout the paper, the term water-wet will be used to refer to hydrophilic systems, while hydrophobic systems will be referred to as oil-wet.

The wettability order is a principal characteristic of three-phase flow, which dictates the size of the pores occupied by each fluid phase [12]. The most wetting phase tends to reside close to the solid surface and in small pores; the most non-wetting phase preferentially fills the centres of the large pores; and the intermediate-wet phase occupies medium-sized pores and/or forms spreading layers sandwiched between the two other phases. The wettability order of a phase can significantly impact its flow conductance; the larger the pores filled by a fluid phase the more readily it flows in the pore space [13]. Furthermore, the existence of a phase in the centre of the pores permits its trapping in the form of disconnected bubbles by the more wetting phases. It has been shown that wettability order is a function of surface wettability and liquid–gas miscibility, either immiscible or near-miscible conditions [12,14,15].

In a system containing oil, gas and water at immiscible gas–oil conditions, the wettability order for water-wet media is water–oil–gas from most to least wetting, with oil spreading in layers sandwiched between gas in the centres and water in the corners of the pore space [16,17]. For strongly oil-wet systems, at immiscible conditions, the wettability order is altered such that oil becomes the most wetting phase, water the most non-wetting, while gas is intermediate-wet; however, gas does not spread in layers at immiscible conditions [18]. In contrast, for oil-wet systems, at near-miscible conditions, gas, the intermediate-wet phase, spreads in layers surrounding water, the most non-wetting phase, in the centres of the pores [15]. Furthermore, in water-wet systems, at near-miscible conditions, the strict wettability order breaks down as oil and gas become neutrally wetting to the rock surface, which prevents the formation of oil layers; water remains the most wetting phase in the pore space [14].

In a three-phase system, the formation of spreading layers maintains hydraulic connectivity in the pore space, permitting flow at very low saturations [19,20]. This is particularly favourable for oil recovery applications, where the injection of gas reconnects oil in the pore space, slowly draining it through oil spreading layers [21].

Layer formation is thermodynamically regulated by the spreading coefficient (*C_s_*) of each fluid phase, which is derived from a force balance involving fluid–fluid interfacial tensions [12]. A phase (*i*) forms spreading layers in the pore space if its spreading coefficient is positive or close to zero (*C_si_* = *σ_jk_* − *σ_ij_* − *σ_ik_*, where *σ* is the interfacial tension and subscripts *i*, *j* and *k* denote the three fluid phases) [22,23]. Øren *et al*. [16] visualized the formation of spreading oil layers sandwiched between water and gas in a water-wet two-dimensional micromodel. Furthermore, Alhosani *et al*. [15] observed the formation of gas spreading layers in an oil-wet carbonate rock under near-miscible conditions. The spreading of a fluid phase can have a direct impact on contact angles and fluid configurations with a major influence on flow properties [12].

Another unique feature of three-phase flow is double and multiple displacement events, which can occur under capillary-dominated conditions [16,20,21,24]. Multiple displacements refers to the displacement of one fluid phase by another, which in turn displaces another phase in the pore space, with any number of intermediate steps [24–26]. Previous micromodel studies have shown the occurrence of double displacement events in systems with variable wettability [27–29]. In a water-wet micromodel, at immiscible conditions, Keller *et al*. [19] observed the occurrence of double drainage (gas displacing oil displacing water) and double imbibition (water displacing oil displacing gas) events during GI and chase water re-injection, respectively. Notice that the direct displacement of water by gas and gas by water is limited due to the formation of spreading of oil layers sandwiched between gas and water, preventing their direct contact in the pore space [28]. Multiple displacement events have also been observed in water-wet micromodels during cycles of gas and water injection; this behaviour was captured using pore network modelling [25]. Furthermore, Sohrabi *et al*. [27] visualized double displacement events in an oil-wet micromodel at immiscible conditions. They reported that the main double displacement event during GI was gas–oil–water with a modest amount of water–oil–gas displacement during chase water re-injection. The behaviour could also be reproduced using a pore-scale model that incorporated multiple displacement events [26].

Most of the research work conducted to visualize the displacement dynamics during multiphase flow has been on micromodels [11,30,31]. While two-dimensional micromodels are useful for viewing pore-level events owing to their visual clarity, they do not capture the flow behaviour of the fluids in three-dimensional porous media with complex structures, e.g. rocks and soils. In this work, we use synchrotron X-ray micro-tomography to image three-phase (gas, water and oil) displacement dynamics during GI in an oil-wet (hydrophobic) reservoir rock. Our ability to visualize the movement of the fluids and characterize the interactions at their interfaces inside the pore space will help provide an in-depth understanding of the physical processes involved.

The use of X-ray micro-tomography allows for the *in situ* three-dimensional imaging of the rock pore space, and has been employed recently to study multiphase flow in porous media [32–35]. Laboratory-based X-ray micro-tomography, also known as static imaging, allows for the end-states of flooding experiments to be imaged. While static imaging enables the wettability order and presence of spreading layers to be determined, double displacement events can only be inferred from these results [14,17,18,36]. Scanziani *et al*. [17] performed three-phase flow experiments in water-wet carbonates, with static imaging, and confirmed, *in situ*, the spreading of oil layers and the anticipated wettability order: water–oil–gas from most to least wetting. Alhosani *et al*. [14] observed, in the same rock–fluid system used by Scanziani *et al*. [17], that oil layers diminish as gas–oil miscibility is approached (near-miscible conditions). The authors also suggested that, at near-miscible conditions, gas can directly displace water in the pore space, facilitating the occurrence of gas–water–oil double displacement events [14].

Qin *et al*. [37] studied gas and water injection in weakly oil-wet rocks, where water was the intermediate-wet phase. They demonstrated that water does not form spreading layers in the pore space, and that gas was trapped by both oil and water during water injection. Furthermore, using static imaging, Alhosani *et al*. [18] showed that in strongly oil-wet systems, at near-miscible and immiscible conditions, the wettability order becomes oil–gas–water from most to least wetting, preventing the trapping of gas by water in the pore centres. Nonetheless, although static imaging provides useful information on the arrangement of fluids in the pore space, it cannot be used to capture the displacement dynamics, which occur on a much shorter time scale than that required for a single static scan (which can take several minutes or hours).

To directly visualize double displacement events and obtain information about the evolution of the fluid arrangement in the pore space over time, fast synchrotron X-ray micro-tomography can be used, which allows for pore-scale images to be acquired at approximately 1 min temporal resolution [38–42]. The use of synchrotron X-ray imaging has provided valuable insights into the pore-scale dynamics of two-phase flow [38,43–47]; however, very few studies have used it to investigate the displacement events during three-phase flow [48,49]. Scanziani *et al*. [48] were the first to use synchrotron imaging to study three-phase—water, oil and gas (nitrogen)—displacement dynamics in a water-wet quarry limestone rock. The authors observed that gas moves in a connected front surrounded by oil spreading layers during GI. Moreover, they reported that during chase water re-injection, after GI, the dominant displacement event was water displacing oil displacing gas, which resulted in double capillary trapping—gas trapping by oil layers and oil layer trapping by water wetting layers. This is favourable for gas storage applications, where immobilization of the gas phase is desired.

Furthermore, Scanziani *et al*. [49] employed synchrotron imaging to investigate the displacement dynamics in a rock with altered wettability, which displayed a mixed-wet behaviour with oil–water contact angles both above and below 90°. The gas remained largely connected during GI in the mixed-wet rock. The authors did not observe double displacement events during GI and chase water re-injection; both gas and water directly displaced oil in the pore space. This type of displacement can facilitate further oil recovery from petroleum reservoirs with limited gas recycling. Nevertheless, to date, no three-phase flow synchrotron study has been performed in an oil-wet (hydrophobic) porous medium. To place the work in a more general context, an accurate characterization of three-phase flow in hydrophobic systems is important since many natural and engineered surfaces are non-water-wet, or designed to be partially water-wet, from deep oil reservoirs to butterfly wings, human skin, textiles, medical devices and fuel cells [50–55].

In this work, we use synchrotron X-ray imaging, with high spatial and temporal resolutions, to investigate the pore-scale dynamics during immiscible GI in an oil-wet reservoir rock at subsurface conditions (8 MPa and 60°C). This is the three-phase extension of an analysis of two-phase displacement [47] using the apparatus and experimental methodology applied to a quarry carbonate [49]. First, we characterize the fluid–fluid contact angles and pore occupancy to confirm the hydrophobic nature of the rock surfaces and infer the wettability order of the system. Then, we use fast imaging to examine, *in situ*, during GI, the evolution of (i) gas connectivity; (ii) direct, double and multiple displacements, events; (iii) water connectivity and trapping; and (iv) spreading layers. Finally, we quantify the change in Minkowski functionals—fluid saturations, interfacial areas and curvatures—with time to provide a complete description of the fluid topology in the pore space, i.e. fluid–fluid connectivity and trapping.

We observe that gas, the intermediate-wet phase, progresses through the pore space in the form of disconnected clusters. This behaviour is attributed to the pore-scale events, made possible by double and multiple displacements, that govern the gas movement in the porous medium, which we name three-phase Haines jumps. As gas displaces either oil or water, it rapidly progresses to fill several pores, which causes it to retract from regions further away to enable this fast filling. This retraction leads to a permanent disconnection of gas ganglia, which fail to get reconnected as GI proceeds. The disconnected gas ganglia reach a new position of capillary equilibrium in the pore space and can only be displaced through double or multiple displacement events.

The significant new observation is that gas is able to progress through the pore space, under capillary-dominated flow conditions, as disconnected ganglia. This is a process unique to three-phase flow and distinct from ganglion dynamics in two-phase flow [56], where a disconnected phase can advect through the pore space when viscous forces are significant.

## 2. Material and methods

**Figure 1.**
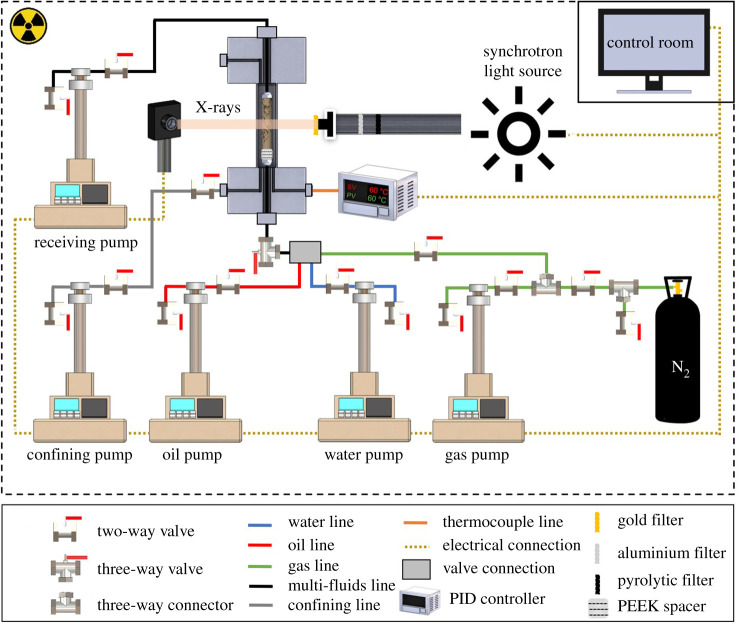
The flooding and imaging apparatus used to conduct the three-phase flow experiment in the oil-wet reservoir rock at 8** **MPa and 60°C. The rock was inserted in a flow cell placed in front of the synchrotron light source to image the movement of water, oil and gas in the pore space. The three fluid phases were injected at a very low flow rate using syringe pumps to capture the pore-scale displacement dynamics. (Online version in colour.)

All synchrotron X-ray imaging was performed using beamline I13-2 at the Diamond Light Source science facility located at the Harwell Innovation Centre, Didcot, UK. The preparation of the experimental materials and fluids as well as wettability alteration process were all conducted in-house before transport to the synchrotron facility to dynamically image the three-phase flow experiment. The methods and apparatus used (figure 1) are similar to those applied to study two-phase waterflooding on the same sample [47] and three-phase flow on a quarry carbonate [49].

### (a) Experimental materials

#### (i) Porous medium properties

The porous medium selected for the three-phase flow study was a heterogeneous carbonate rock extracted from a giant oil-producing reservoir in the Middle East. We only studied a single sample because of constraints on experimental time at the synchrotron facility. The cylindrical sample was 3.85 mm in diameter and 13.8 mm in length. The mineralogical composition of the reservoir rock consisted of mainly calcite (96.5% ± 1.9%) [57].

The total porosity (ratio of the volume of the void space to the total volume) was measured using a helium porosimeter to be 26%. The total porosity is composed of macro- and micro-porosities. The macro-porosity is defined as the porosity that is resolvable from the pore-scale images, while the micro-porosity is the unresolvable porosity. The macro- and micro-porosities accounted for 16% and 10%, respectively. The total pore volume (PV) of the sample corresponding to the measured helium porosity was 0.0416 ml.

#### (ii) Fluid properties

**Figure 2.**
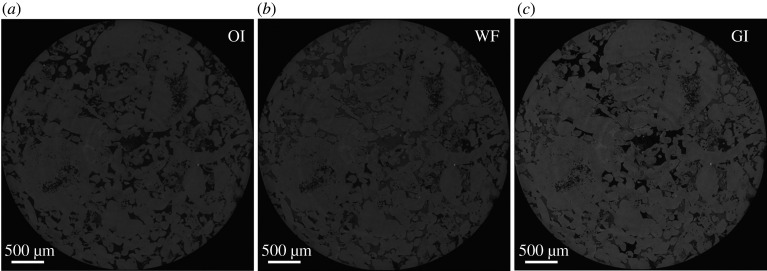
Filtered raw two-dimensional pore-scale images of a cross-section of the rock acquired after: (*a*) oil injection (OI); (*b*) water flooding (WF); and (*c*) gas injection (GI), with a voxel size of 3.5** **µm. In (*a*), rock is the light phase and oil is the dark phase. In (*b*) and (*c*), the order from darkest to brightest phase is oil–water–rock and gas–oil–water–rock, respectively.

The three fluids, two immiscible liquids and a gas, used in the experiment consisted of deionized water, *n*-decane and nitrogen. To distinguish between the oil phase (*n*-decane) and the water phase (deionized water) in the raw pore-scale images *n*-decane was doped with 15 wt% 1-iododecane (C_10_H_21_I), while deionized water was doped with 20 wt% potassium iodide (KI). This provided a distinct X-ray attenuation for each phase in the pore-scale images, facilitating accurate segmentation. The order of grey-scale values in the pore-scale images, from lowest to highest (darkest to brightest), was gas–oil–water–rock; see figure 2. The thermophysical properties of the three fluid phases are listed in table 1.

**Table 1.** Densities, viscosities, interfacial tensions and spreading coefficients of the three fluid phases used in the three-phase flow experiment conducted at 8** **MPa and 60°C. The spreading coefficient of each phase, *i*, was calculated using *C_s__i_* = *σ_jk_* − *σ_ij_* − *σ_ik_*, where *σ* is the interfacial tension and subscripts *i*, *j* and *k* denote the three fluid phases. The interfacial tensions were measured using the pendant drop method under the experimental conditions (8** **MPa and 60°C) [58]. Densities were measured at 40°C and 7.6** **MPa. The viscosity of *n*-decane is provided at ambient conditions [59], and of water and nitrogen at 50°C and 10** **MPa [60].

fluid	composition (%wt)	*ρ* (kg · m^−3^)	*µ* (mPa · s)	*σ* (mN · m^−1^)	*C_s_* (mN · m^−1^)
water	0.80 H_2_0 + 0.20 KI	1154.1	0.547	*σ_gw_* = 63.7	−104.6
oil	0.85 C_10_H_22_ + 0.15 C_10_H_21_I	715.2	1.088	*σ_ow_* = 52.1	+0.4
gas	N_2_	83.9	0.018	*σ_go_* = 11.2	−22.8

The interfacial tensions between the fluids, water (80 wt% H_2_O + 20 wt% KI), oil (85 wt% C_10_H_22_ + 15 wt% C_10_H_21_I) and nitrogen were measured under the experimental conditions, 8 MPa and 60°C, using the pendant drop method. The apparatus for conducting the interfacial tension measurements is described elsewhere [58]. Using the values in table 1, the spreading coefficients of the three fluid phases were calculated to be *C_sw_* = −104.6 mN m^−1^, *C_so_ *= +0.4 mN m^−1^ and *C_sg_* = −22.8 mN m^−1^. This indicates that it is only possible for oil to form spreading layers in the pore space since its spreading coefficient is close to zero; water and gas do not spread in layers. Gas, indeed, did not form layers in these experiments, unlike in near-miscible systems, where the spreading coefficient is closer to zero and gas layers can be seen [15].

### (b) Establishing hydrophobic wettability

The wettability of the rock surfaces was altered towards hydrophobic conditions using a process known in the oil industry as ageing [61,62]. Ageing is a chemical process in which the solid surfaces are exposed, at high temperatures and pressures, to crude oil components that can be adsorbed by the rock surface, which reverts its wettability from water-wet to oil-wet. In this study, the crude oil used to alter the wettability was obtained from the same reservoir as that from which the rock was extracted. The composition of the crude oil is listed in electronic supplementary material, table S1. It is believed that the original underground wettability of the reservoir rock is oil-wet, and hence its wettability can easily be restored to oil-wet conditions.

First, the pressure and temperature of the system were raised to 10 MPa and 80°C to establish the wettability alteration conditions. Then, the rock pore space was saturated with brine, the aqueous phase from the same reservoir, which was then followed by the injection of 40 PV of crude oil from the top and bottom of the sample with an increasing flow rate from 0.001 to 0.1 ml min^−1^. After that, 5 PV of fresh crude oil was injected in the rock at 0.05 ml min^−1^ each day for a week. Finally, the rock was conserved in a crude oil bath at ambient pressure and 80°C for three months before transporting it to the synchrotron facility to conduct the experiment.

### (c) Experimental procedure

Subsequent to preparing the experimental materials and altering the surface wettability of the porous medium towards hydrophobic conditions, the three-phase flow experiment was performed at beamline I13-2. The flooding apparatus was set up at the synchrotron beamline to image the flow of fluids in the rock pore space with high temporal (74 s) and spatial (3.5 µm) resolutions.

#### (i) Apparatus

A high-pressure, high-temperature flooding apparatus was used to perform the three-phase flow experiment at 8 MPa and 60°C. A schematic diagram of the experimental apparatus is shown in figure 1. The apparatus consisted of the following parts.

*Pumps.* High accuracy, low flow rate Teledyne ISCO pumps were used to regulate the flow of the fluids through the rock sample.

*Flow cell.* A Hassler-type carbon fibre coreholder that is X-ray transparent was used to keep the rock under confining pressure during the experiment.

*Synchrotron light source.* The carbon fibre coreholder was placed in front of a high photon flux pink beam emitted by the synchrotron light source to image the rock and fluids during the experiment. The X-ray beam had a peak photon energy of 15 keV and was filtered by placing a 1.3 mm pyrolytic carbon filter, a 3.2 mm aluminium filter and a 10 µm gold filter in the beamline.

*Proportional integral derivative (PID) controller.* A PID controller, connected to a flexible heater wrapped around the flow cell, was used to elevate the temperature of the rock to the experimental conditions. In addition, a thermocouple, placed next to the sample, was also connected to the PID controller to maintain and regulate the temperature during the experiment.

*PEEK spacer.* An X-ray-transparent spacer made of polyetheretherketone (PEEK) was placed at the inlet of the rock sample to detect the arrival of the fluids in order to start the dynamic X-ray imaging.

#### (ii) Flow experiment

A series of fluid injections: (i) oil injection (OI), (ii) water flooding (WF), and (iii) GI, were performed in the aged reservoir rock, during which the pore space was continuously imaged to capture the dynamics of displacement. All injections were performed from the bottom of the sample under capillary-dominated conditions; see table 2. Figure 2 shows two-dimensional raw pore-scale images of a cross-section of the rock acquired after each injection step.

**Table 2.** Details of the fluid injections performed during the three-phase flow experiment at 8** **MPa and 60°C. Pore volumes (PV) injected correspond to the total porosity of the rock sample. WF and GI were stopped when no significant change in the fluid configurations in the pore space had been observed for at least 15 min. Capillary numbers were calculated using *Ca *= *µq*/*σ*, where *σ* is the interfacial tension, *µ* is the viscosity of the injected fluid and *q* is the Darcy velocity. Subscripts *w*, *g* and *o* stand for water, gas and oil phases, respectively. *σ* and *µ* are shown in table 1, while *q* is calculated by dividing the flow rate by the cross-sectional area of the sample (11.34 mm^2^).

injection step	flow rate (ml min^−1^)	PV	total time (min)	capillary number
oil injection (OI)	0.1	20.0	8.32	—
water flooding (WF)	0.00015	0.69	92.1	*Ca*_[*wo*] _= 2.09 × 10^−9^
gas injection (GI)	0.00015	0.24	32.2	*Ca*_[*go*] _= 3.64 × 10^−10^
				*Ca*_[*gw*] _= 6.39 × 10^−11^

First, 20 PV of oil (doped *n*-decane) was injected into the sample at a flow rate of 0.1 ml min^−1^ to replace all of the crude oil used to alter the wettability of the sample; see figure 2*a*. The temperature and pressure of the system were then raised to the experimental conditions (60°C and 8 MPa), and a confining pressure of 10 MPa was applied. Water injection (WF) was then started at a very low flow rate, 0.15 µl min^−1^, corresponding to a capillary number *Ca*_[*wo*]_ of 2.09 × 10^−9^, defined by *Ca *= *µq*/*σ*, where *σ* is the interfacial tension, *µ* is the viscosity of the displacing fluid and *q* is the Darcy velocity (table 1). Water was injected over a period of 92.1 min, which corresponded to the injection of 0.69 PV of water; see figure 2*b*. GI was then performed at the same flow rate for 32.2 min, corresponding to the injection of 0.24 PV of gas, with a gas–water *Ca*_[*gw*]_ = 6.39 × 10^−11^ and gas–oil *Ca*_[*go*]_ = 3.64 × 10^−10^. WF and GI were stopped when no significant change in the fluid configurations in the pore space had been observed for at least 15 min.

#### (iii) Synchrotron X-ray imaging

**Figure 3.**
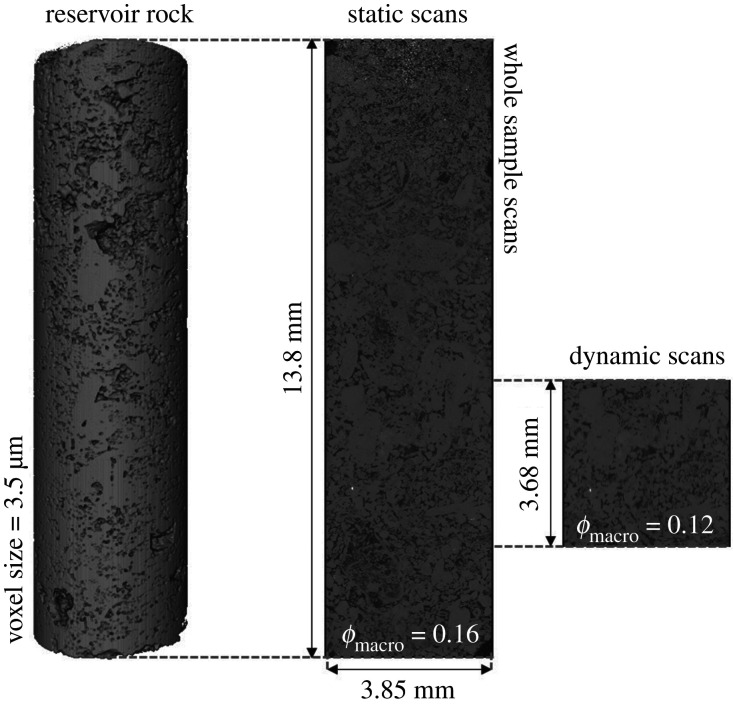
Three-dimensional images showing the location of the dynamic scans (1280 × 1280 × 1080 voxels) relative to the static scans of the whole sample (1280 × 1280 × 3940 voxels). The spatial resolution of the images is 3.5** **µm. The macro-porosities (*ϕ*_macro_) of the static and dynamic scans are 16% and 12%, respectively.

Static and dynamic scans were collected during the experiment with a voxel size of 3.5 µm. The dynamic scans were acquired during the injection of fluids, whereas static scans were acquired at the end of each injection. Dynamic imaging was performed at the middle of the sample, in the vertical direction, while static imaging of the whole sample was performed. The location of the dynamic scans relative to the static scans is shown in figure 3. The centre of the sample was selected for dynamic imaging since it does not contain large vugs and mineral grains. The macro-porosity is 16% in the static scans, as mentioned in §2a(i), and 12% in the dynamic scans.

The dynamic images were 1280 × 1280 × 1080 voxels in size. During dynamic imaging of water injection, a total of 76 tomograms were acquired, every 70 s, with 700 projections and 0.065 s exposure time. On the other hand, 25 tomograms were acquired, every 74 s, during GI with 750 projections and 0.07 s exposure time. The high spatial and temporal resolution of synchrotron imaging allowed for the pore-scale displacement dynamics to be captured during water and GIs. The static scans of the whole sample were acquired after each injection (see electronic supplementary material, figure S1) with 2000 projections and 0.15 s exposure time.

### (d) Image and data processing

#### (i) Image segmentation

All the tomograms acquired were reconstructed using a filtered back-projection algorithm [63,64] obtaining grey-scale images of the pore space and the fluids within it, as shown in figure 2 and electronic supplementary material, figure S1. However, to obtain quantitative information from these images they must be segmented. Segmentation refers to the assignment of voxels to each phase—water, oil, gas or rock—in the pore-scale image. The large static images were segmented using the seeded watershed algorithm [65], while the dynamic images were segmented using machine learning-based Weka segmentation [66,67].

Segmentation of the static images was performed in three steps. (i) The images acquired after OI, WF and GI were filtered using a non-local means filter [68]. (ii) The filtered WF and GI images were then subtracted from the filtered OI image to clearly distinguish the water and gas phases in these images. (iii) The subtracted images were then filtered again with the non-local means filter and segmented using the watershed algorithm. The same procedure was followed to segment the dynamic WF and GI images; however, Weka was used instead of watershed since it provides a more accurate characterization of flow properties near fluid–fluid contacts [57]. During Weka segmentation the fast-random algorithm was selected alongside the mean and variance texture filters. Weka segmentation is shown in electronic supplementary material, figure S2. Weka is very CPU intensive, which explains why it was not applied to segment the large static images.

#### (ii) Data analysis

*Contact angle measurements.* The segmented three-dimensional pore-scale images can be used to characterize the *in situ* geometric contact angles, *θ_g_*, between the three fluid phases in the pore space. We use the automatic code developed by AlRatrout *et al*. [69] to measure the oil–water, gas–water and gas–oil contact angles. The geometric oil–water contact angle can be used to infer the wettability of the surface [57]; rock surfaces can either be water-wet or oil-wet, defined by the oil–water contact angle. Nonetheless, geometric contact angles are measured between relaxed fluid interfaces, when fluids are at mechanical equilibrium, and may not be representative of the actual contact angle value during displacement.

To complement the geometric contact angle measurements, we use an energy balance approach to calculate fluid–fluid displacement contact angles, also known as three-phase thermodynamic contact angles (*θ_t_*), between oil–water, gas–water and gas–oil [12]. Thermodynamic contact angles have been proven to provide better estimates of displacement angles in two-phase flow than geometric values [46,47,70]. Assuming no change in Helmholtz free energy between the two local states of equilibrium and ignoring viscous dissipation, the three-phase thermodynamic contact angles can be calculated using [71]

2.1 $$(\Delta a_{ws}\cos\theta_{t[ow]}-\Delta a_{ow}-\phi \kappa_{ow}\Delta S_{w})\sigma_{ow}=(\Delta a_{gs}\cos\theta_{t[go]}+\Delta a_{go}-\phi \kappa_{go}\Delta S_{g})\sigma_{go}+\Delta a_{gw}\sigma_{gw},$$

where *a* is the interfacial area per unit volume, *θ_t_* is the thermodynamic contact angle, *ϕ* is the dynamic image-based macro-porosity, *S* is the saturation (the fraction of the macro-pore space occupied by each phase) and *κ* is the total curvature of the fluid–fluid interface. Subscripts *s*, *w*, *g* and *o* denote the solid, water, gas and oil phases, respectively, while Δ is the change between two consecutive time steps.

The interfacial areas, curvatures and saturations were measured on the 25 dynamic pore-scale images obtained during GI, and the values of *θ_t_*_[*ow*]_ and *θ_t_*_[*go*]_ that best fit equation (2.1) were found using the least-squares approximation approach. The third contact angle, *θ_t_*_[*gw*]_, was found using the Bartell–Osterhof relationship for three phases in thermodynamic equilibrium [72,73],

2.2 $$\sigma_{gw}\cos\theta_{gw}=\sigma_{go}\cos\theta_{go}+\sigma_{ow}\cos\theta_{ow}.$$

*Saturation, specific interfacial area and curvature.* The saturations of the three fluid phases, that is, the ratio of the volume of a phase to the volume of the pore space, were computed on the static and dynamic images by dividing the number of voxels assigned to each phase by the total number of voxels comprising the pore space in the segmented images. This only considered saturation in macro-pore space (the resolvable pores in the image).

The specific interfacial area and curvature of the fluid–fluid interfaces were measured on the segmented dynamic pore-scale images. First, a marching cubes algorithm was used to isolate the gas–water, gas–oil and oil–water interfaces. The interfaces were then smoothed, to remove voxelization artefacts, using unconstrained smoothing with a kernel size = 5 [74,75]. The specific interfacial area is the area of these interfaces divided by the total volume—that is, the volume of the rock and pore space combined. To obtain the fluid–fluid curvatures, a further step was required where the smoothed interfaces were additionally modelled using a quadratic equation, whose eigenvalues and eigenvectors correspond to the principal curvatures (*κ*_1_ and *κ*_2_) and their directions, respectively.

In addition to facilitating the calculation of thermodynamic contact angles, obtaining quantitative information on saturation, interfacial area and principal curvatures—these properties are also known as Minkowski functionals—can provide a complete topological description of the geometry of the fluids within the pore space [76–78]. This information can help understand key physical characteristics of flow, such as fluid–fluid connectivity and trapping. For instance, the product of the two principal curvatures of the fluid–fluid interface (*κ*_1_ × *κ*_2_), also known as the Gaussian curvature, can be used as a measure of the connectedness of the fluid phases in the pore space [78]. Furthermore, the sum of the two principal curvatures—the total curvature (*κ* = *κ*_1_ + *κ*_2_)—can be linked to the capillary pressure (*P_c_*) between the fluids [79], which is the pressure needed for one phase to displace another in the pore space, using the Young–Laplace equation

2.3 $$P_{c,ij}=\sigma_{ij}\kappa_{ij}.$$

*Pore occupancy, connectivity and thickness maps.* Pore occupancy—the size of the pores occupied by each fluid phase—was characterized using the maximal ball method [80,81], which relies on the generalized pore network extraction code [82]. First, the size of the pores was determined by fitting the largest inscribed spheres in their centres; the diameter of the sphere is the diameter of the pore. The fluid phase that resides in the centre of the sphere—the centre of the pore—is considered to occupy the pore. This allows us to quantitatively assess the relationship between the pore size and the phase occupying it. We quantify the pore occupancy on the static images after WF and GI.

The three-dimensional connectivity of each fluid phase was examined in the dynamic scans. The voxels belonging to each phase were isolated and then the connectivity analysis was performed. Each voxel in an individual object is assigned an identical value, thereby labelling the disconnected clusters with distinct colours. The thickness maps of a phase, defined as the diameter of the largest ball containing the voxel and entirely inscribed in the object, were computed in three dimensions using the approach developed by Hildebrand & Rüegsegger [83].

## 3. Results and discussion

First, in §3a, we measure fluid–fluid contact angles to confirm that the ageing process altered the wettability of the rock surfaces towards hydrophobic conditions. We use the geometric and thermodynamic contact angle measurements alongside pore occupancy to identify the wettability order of the system. Next, using static images of the whole sample, we show the end-state saturations of oil, water and gas after each injection in §3b. In §3c, we analyse the GI dynamics by examining the evolution of (i) gas connectivity; (ii) direct, double and multiple displacement events; (iii) water connectivity and trapping; and (iv) oil layers. Finally, in §3d, we quantify the change in Minkowski functionals—saturations, interfacial areas and curvatures—with time to obtain a complete understanding of the fluid topology in the pore space.

### (a) Wettability characterization

#### (i) Contact angles

**Figure 4.**
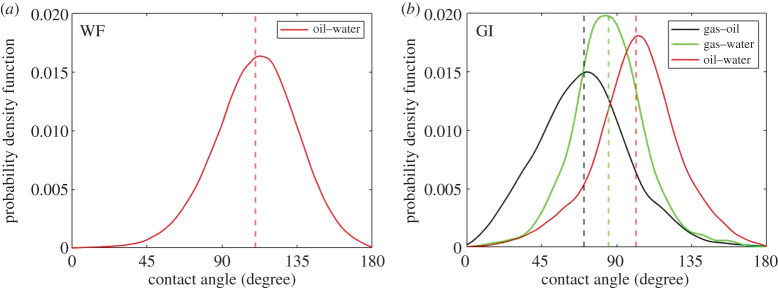
Probability density function of the *in situ* measured distribution of fluid–fluid contact angles at the end of (*a*) waterflooding (WF) and (*b*) gas injection (GI). The contact angles were measured using the automated method developed by AlRatrout *et al*. [69]. The angle was characterized through the denser phase: water in the case of oil and water and gas and water, and oil in the case of gas and oil. (Online version in colour.)

The geometric fluid–fluid contact angles were measured at the end of WF and GI, in the same location, on a subvolume of size 0.5 × 0.5 × 0.5 mm^3^. Figure 4 shows the *in situ* spatial distribution of the effective oil–water, gas–water and gas–oil contact angles after WF and GI.

After WF, the mean geometric oil–water contact angle was 110 ± 20°, indicating that oil is more wetting to the rock than water, and hence confirms that the ageing process rendered the rock surfaces oil-wet (hydrophobic); see figure 4*a*.

Furthermore, figure 4*b* shows that the mean oil–water contact angle decreases to 101 ± 22° after GI (table 3). In three-phase flow, double displacement mechanisms allow for both water to displace oil and oil to displace water in the pore space. In the latter process, water is receding, with a likely lower contact angle than the advancing angle during WF, because of contact angle hysteresis. Therefore, the mean geometric contact angle decreases after GI, representing a position of equilibrium after events where water is both invading and receding.

**Table 3.** Measurements of the oil–water, gas–oil and gas–water mean geometric contact angles and thermodynamic contact angles after gas injection (GI). The error in the geometric contact angle represents the standard deviation of the distribution, while in the case of the thermodynamic contact angle it indicates the uncertainty in the measurements.

method	*θ_ow_*	*θ_go_*	*θ_gw_*
geometric	101 ± 22°	70 ± 27°	87 ± 25°
thermodynamic	125 ± 10°	78 ± 10°	115 ± 10°

The mean geometric gas–oil contact angle is 70 ± 27° (figure 4*b*), once more indicating that oil is more wetting to the surface than gas; therefore, it is the most wetting phase in the system. The measured mean of the gas–water geometric contact angle distribution is 87 ± 27°, suggesting that the rock surfaces are neutrally wetting to both gas and water. Hence, it is not possible to determine a clear wettability order in the system using the geometric contact angle measurements only, which record values on hinging contact lines rather than the angles during a displacement.

To characterize the fluid–fluid contact angles encountered during displacement, we use equation (2.1) to find the gas–oil *θ_t_*_[*go*]_ and oil–water *θ_t_*_[*ow*]_ thermodynamic angles that best fit the data using the least squares approach; see the electronic supplementary material. The thermodynamic contact angle calculations yield an oil–water angle of 125 ± 10° and a gas–oil angle of 78 ± 10°; see table 3. The gas–water thermodynamic contact angle is determined using equation (2.2) as 115 ± 10°. While the interpretations of the oil–water and gas–oil contact angles in this analysis are broadly consistent with those of the geometric contact angle measurements, the thermodynamic gas–water contact angle suggests that gas is, on average, more wetting to the rock than water. This allows us to establish a clear wettability order in the system, one in which oil is wetting to both water and gas, gas is non-wetting to oil and wetting to water, while water is non-wetting to both oil and gas. This implies that water will tend to occupy the larger pores and that the gas–water capillary pressure will be negative, as we will show later.

**Figure 5.**
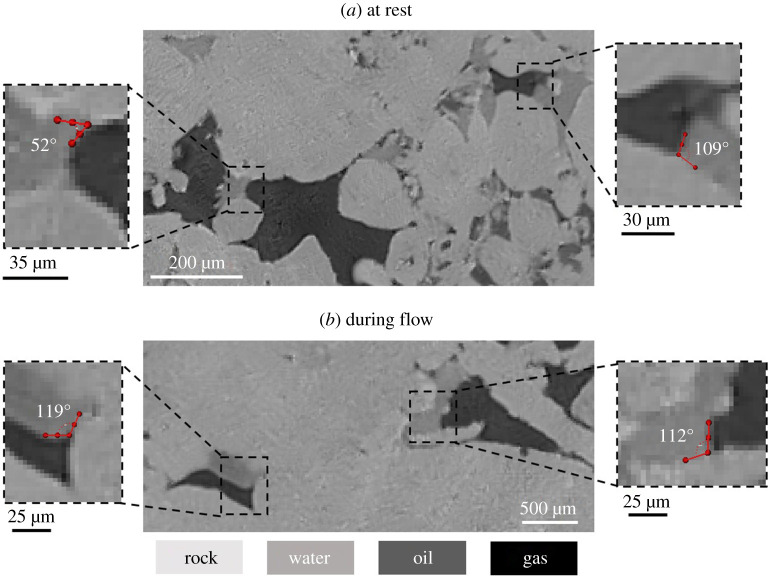
Two-dimensional raw pore-scale images, with a voxel size of 3.5** **µm, showing (*a*) the contact angles formed between gas and water when the fluids are at rest and (*b*) the gas–water contact angles during the displacement of water by gas. (Online version in colour.)

From table 3, we observe that the geometric contact angle tends to underestimate the displacement contact angles. This is further illustrated in figure 5 by visually inspecting gas–water contacts on static and dynamic raw pore-scale images. At rest, water forms contact angles with gas that are both lower and larger than 90°, indicating that the rock surfaces are neutrally wetting to gas and water. On the other hand, during the displacement of water by gas, we notice that the gas–water contact angle is almost always larger than 90°, implying that gas is wetting to water during flow (figure 5). Moreover, this behaviour was also seen in a recent modelling study, where the use of the geometric contact angle was insufficient to match experiments of WF in rocks with altered wettability; instead, a larger advancing contact angle was needed to match the results [70]. This analysis identifies a clear limitation with the geometric contact angle measurement and shows that it is not representative of displacement contact angles in systems with altered wettability. In contrast, the gas–water thermodynamic contact angle measurement (table 3) is in agreement with the angles observed during gas–water displacement (figure 5), indicating that it is more representative of displacement angles than the direct geometric measurement.

#### (ii) Pore occupancy

**Figure 6.**
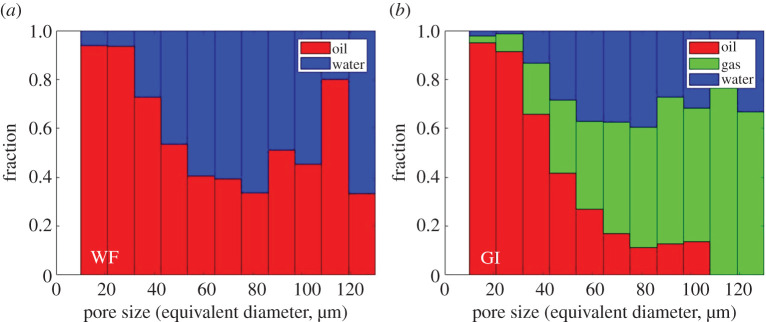
Normalized bar charts showing the pore occupancy in the oil-wet rock, characterized on static images of the whole sample, after (*a*) water flooding (WF) and (*b*) gas injection (GI). (Online version in colour.)

To further confirm the wettability order of the system, we quantified the pore occupancy on static images of the whole sample after WF and GI (figure 6). As anticipated, during water injection in an oil-wet system, water displaces oil from the larger-sized pores, confining it to smaller pores (figure 6*a*). Furthermore, figure 6*b* shows that, after GI, water resides in the largest pores, oil the smallest, while gas occupies pores of intermediate size. This confirms that the wettability order of the system is oil–gas–water from most to least wetting. The wettability order inferred from pore occupancy is in agreement with the interpretations of the thermodynamic contact angle measurements. This wettability order has been previously observed in micromodels [29] and laboratory X-ray imaging experiments with CO_2_ in the same reservoir rock [15], but not before with nitrogen as the gas phase.

While in figure 6 there is a tendency for oil to reside in the smaller pores and water in the larger ones, we do still observe gas and water occupancy in pores of all size: there is not a strict segregation. We will discuss this further when we discuss the dynamics of gas invasion, but it is important to note that gas does not have a strong preference for either larger or smaller pores.

### (b) Fluid saturations

The saturations of oil, gas and water in the macro-pore space of the rock were measured on static images of the whole sample after water and GI; see table 4 and electronic supplementary material, figure S3. At initial conditions, the oil and water saturations were 99% and 1%, respectively, measured in the macro-pore space; we presume that water is also initially present in the micro-pores of the rock. After WF, only 48 ± 5% of the oil was recovered. This is ascribed to the oil-wet nature of the rock, where water displaces oil in the centre of the pores only; oil remains connected in thick wetting layers. GI displaces both oil and water out of the pore space; gas displaces 30 ± 5% of the resident oil, while only 16 ± 5% of water is displaced out of the system. A high remaining water saturation indicates that water gets trapped in the pore space of the rock. This is attributed to (i) water being the most non-wetting phase, and hence it remains preferentially in the larger pores (figure 6) and (ii) the preferential displacement of oil by gas in the smaller-sized pores. The gas saturation reaches only 24 ± 5%, which is similar to saturation values observed on the same reservoir rock previously during unsteady-state flooding [15].

**Table 4.** Water, oil and gas saturation in the macro-pore space of the rock after each flooding step. Saturations were measured on the static images of the whole sample. The uncertainty in the measurements is ±0.05.

injection sequence	water saturation	oil saturation	gas saturation
oil injection (OI)	0.10	0.99	—
water flooding (WF)	0.48	0.52	—
gas injection (GI)	0.40	0.36	0.24

### (c) Three-phase flow dynamics

**Figure 7.**
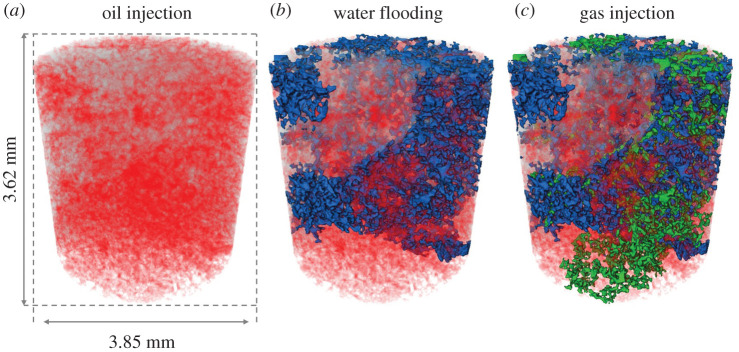
A three-dimensional volume rendering of the fluid configurations in the section of the rock imaged dynamically during (*a*) oil injection, (*b*) water flooding and (*c*) gas injection. Oil is shown in red, water in blue and gas in green. (Online version in colour.)

In this section, we examine the various pore-scale dynamics observed during GI in our oil-wet rock, where the wettability order is oil–gas–water from most to least wetting. The two-phase displacement dynamics encountered during WF will be briefly described to set the scene for the discussion of GI dynamics. A complete description of WF dynamics is provided by Alhosani *et al*. [47]. The pore-scale dynamics were investigated by imaging the rock section shown in figure 7, with a high temporal resolution during WF and GI.

The main finding of this section is that gas moves through the pore space as disconnected clusters through double and multiple displacements; this is a distinct dynamic not seen in two-phase flow, where the injected phase needs to remain connected to progress through the porous medium.

#### (i) Water flooding

During WF, the displacement of oil by water is all piston-like; see electronic supplementary material, movie S1. Water advances as a connected front in an invasion percolation process, where throats, the restrictions between pores, fill in order of size, with the largest available throats filled first; displacement is predominantly size-controlled. This is attributed to the wide pore size distribution of the heterogeneous rock selected. Furthermore, we observe drainage-associated pore-filling dynamics including Haines jumps and snap-off events.

Figure 7*b* shows the fluid configurations in the oil-wet rock at the end of WF—water is shown in blue and oil in red. As anticipated, there is a high remaining oil saturation owing to the strongly oil-wet nature of the rock; not only does oil remain connected in thick wetting layers but also many oil-filled pores have been completely bypassed by the incoming water front as a result of inadequate water pressure to overcome the high oil–water capillary pressure. This is in contrast with observations made in water-wet porous media, where water spontaneously imbibes through wetting layers and corners of the pore space, trapping oil in the centres; no oil-filled pores were bypassed [28]. The dynamics of WF stopped after the injection of 0.58 PV of water (78.1 min).

#### (ii) Gas injection

**Figure 8.**
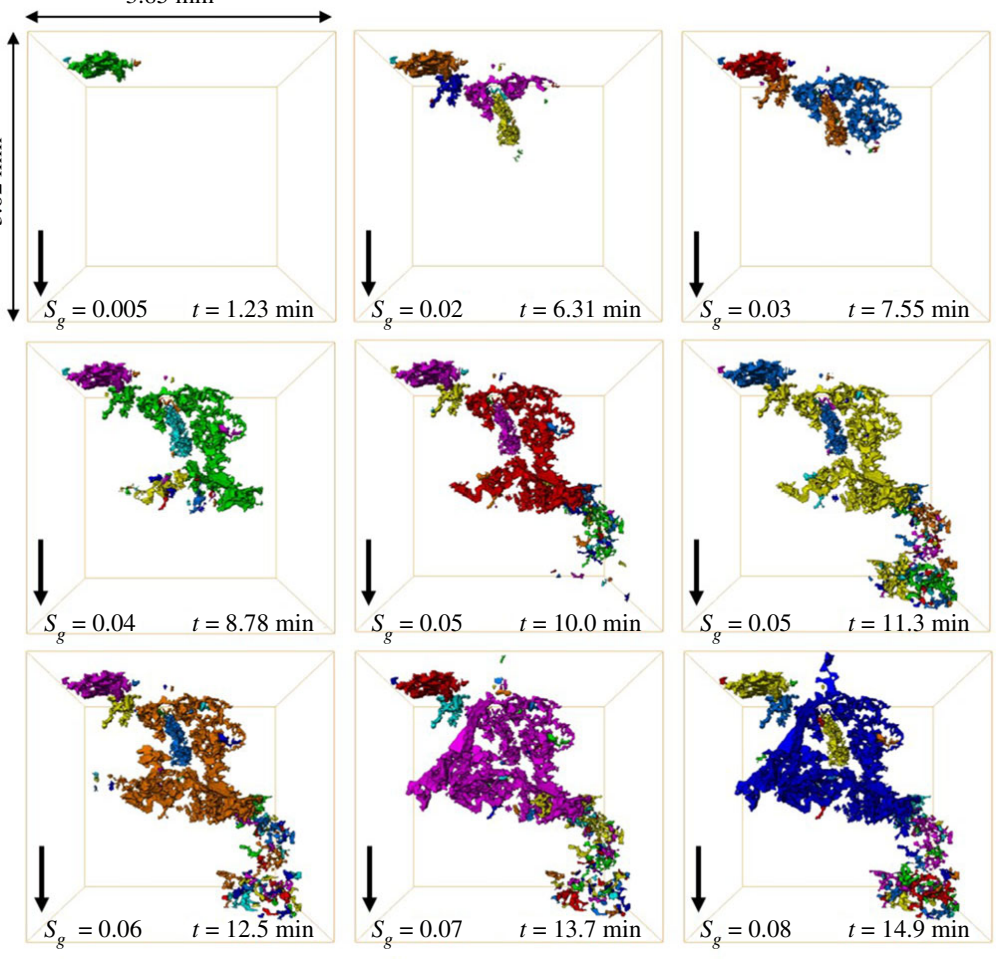
Three-dimensional maps of the gas connectivity in the pore space during GI shown at different time steps. Each disconnected gas cluster is labelled with a different colour. The black arrow points towards the direction of flow. *S_g_* is the gas saturation in the imaged section, while *t* is time. (Online version in colour.)

*Invasion pattern and displacement events*. We observe a distinct three-phase invasion pattern during GI in the oil-wet pore space; see electronic supplementary material, movie S2. Gas, the intermediate-wet phase, advances through the porous medium in disconnected clusters; gas is not connected during GI. The connectivity of gas during GI is captured using dynamic imaging; see figure 8—each colour represents a different gas cluster. This is different from the invasion pattern observed during the two-phase WF in §3c(i).

In two-phase flow, when a non-wetting phase displaces the wetting phase, Haines jumps are observed, which involve the rapid filling of multiple pores followed by retraction and disconnection of the non-wetting phase and the phases come to a new position of equilibrium [38,41]. However, as injection proceeds, the non-wetting phase gets reconnected in the pore space: for capillary-dominated flow, the gas has to reconnect to progress further through the pore space. Haines jumps have been seen during two-phase flow in both water-wet and oil-wet rocks [38,47].

We observe a similar phenomenon during GI under three-phase conditions, namely the filling of several pores by gas, accompanied by the retraction of gas from other regions, leading to disconnection of gas ganglia in the pore space. Nevertheless, unlike two-phase flow, the occurrence of this phenomenon in our three-phase system leads to a permanent gas disconnection; gas does not reconnect as GI proceeds. The disconnected gas ganglia reach a new position of equilibrium in the pore space; gas can only be further mobilized through double and multiple displacement events. This pore-scale phenomenon, which we call a three-phase Haines jump, controls the movement of gas in the pore space; gas displaces oil and water in a sequence of three-phase Haines jumps. The other distinction from two-phase flow is that the gas is intermediate-wet—it is non-wetting to oil but wetting to water.

This phenomenon, three-phase Haines jumps, has not been seen before during three-phase flow in porous media. Previous three-phase synchrotron studies in water-wet and mixed-wet rocks observed that gas progresses in a connected front, maintaining its connectivity in the pore space [48,49]. In these experiments, the gas was either the most non-wetting phase or almost neutrally wet with respect to water. However, a recent study conducted using static imaging, in the same reservoir rock and under immiscible oil-wet conditions, observed that gas was highly disconnected at the end of GI [18]. The authors attributed this to interface relaxation as gas tries to reach a new position of capillary equilibrium in the pore space when GI is terminated; however, using dynamic imaging we deduce that the origin of the poor connectivity is the advance of gas through three-phase Haines jumps.

A similar behaviour to three-phase Haines jump was seen during GI in an oil-wet micromodel [29] that was successfully modelled by considering multiple displacement events [26]. The behaviour was attributed to water blocking. In some cases, the progress of the advancing gas front was blocked if faced with a water-occupied throat (restriction in the pore space). However, as the gas pressure built up, exceeding that of the gas–water capillary pressure, the throat would momentarily open, allowing gas to escape towards the next oil-filled pore. As gas pressure dropped, after displacement, the throat would again be filled by water, disconnecting the gas phase.

**Figure 9.**
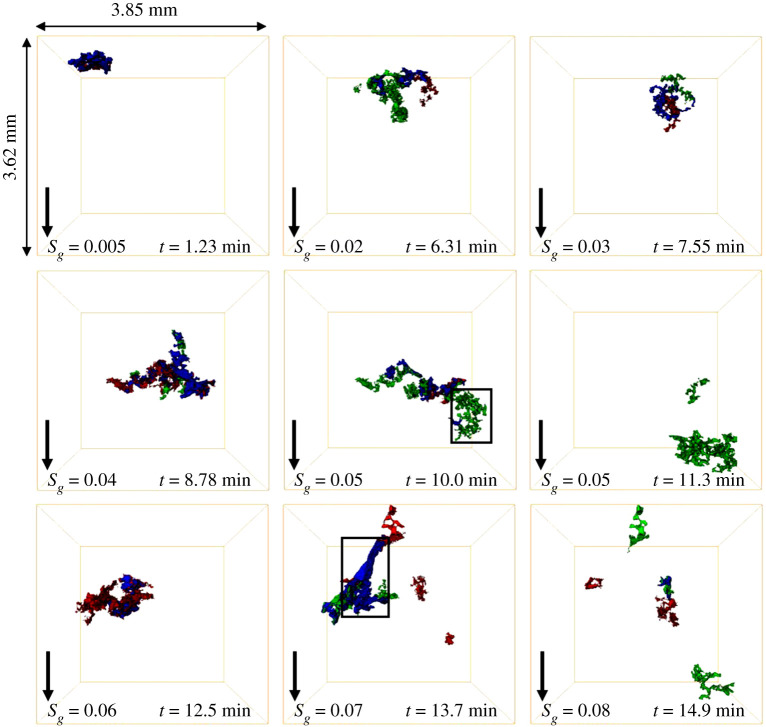
Three-dimensional images of direct and multiple displacement events occurring at different time steps during gas injection in the oil-wet rock. Displacement of oil by gas is shown in green, water by gas in blue and water by oil in red. The black arrow points towards the direction of flow. *S_g_* is the gas saturation in the imaged section, while *t* is time. (Online version in colour.)

Three types of displacement were observed during GI: (i) direct gas–oil displacement; (ii) direct gas–water displacement; and (iii) gas–oil–water double and multiple displacements. Figure 9 shows images of the various displacement events occurring at different time steps—green represents the displacement of oil by gas, blue is water by gas, while red is water by oil. As discussed above, double and multiple displacements [24–26] are necessary to allow gas to propagate in disconnected clusters: in particular, for gas to remain disconnected there must be multiple displacement events of the form gas–oil–gas–water, where the second gas displacement in the sequence involves a trapped cluster. We suspect that there is a thin oil layer surrounding the gas phase during the direct gas–water displacement owing to the positive initial oil spreading coefficient (+0.4 mN m^−1^; table 1); however, it is not visible at the given spatial resolution of the experiment (3.5 µm).

Notice that gas directly displaces oil and water in the pore space, and there is no strong preferential displacement of oil over water as seen in the carbonate rock study with a mixed-wet behaviour [49], where gas only displaced oil in a piston-like displacement during GI; there was no displacement of water by gas. Furthermore, this is different from water-wet systems, where gas only initially displaces water until it comes into contact with oil, which spreads in layers between gas and water, preventing their direct contact in the pore space [48].

Initially, direct, double and multiple displacement events occur close to the advancing gas front; however, after gas breakthrough in the imaged rock section (11.3 min), the pore-scale displacement dynamics continue to occur but at locations throughout the sample. The GI dynamics stop after the injection of 0.18 PV of gas (19.8 min); the gas pressure is insufficient for additional displacement.

**Figure 10.**
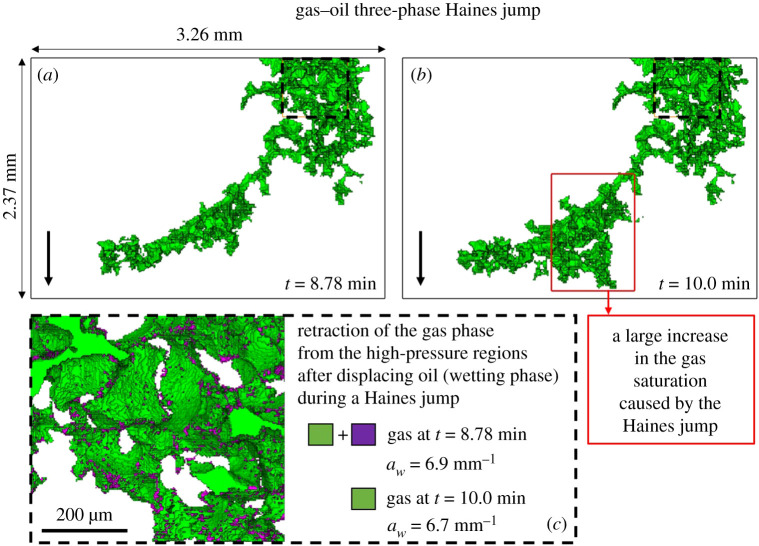
Three-dimensional images of the gas phase at different time steps illustrating the occurrence of a three-phase Haines jump during the displacement of oil by gas in the oil-wet pore space. (*a*) and (*b*) show the difference in gas saturation before and after the three-phase Haines jump. (*c*) The specific interfacial area between gas and the rest of the phases (water, oil and solid) is lower in the high-pressure region, marked by the dashed line, after the three-phase Haines jump owing to gas retraction. The black arrow points towards the direction of flow. (Online version in colour.)

To illustrate the displacement dynamics in more detail, figure 10 shows the rapid filling of multiple pores during a gas-oil three-phase Haines jump, where gas displaces oil overall—this displacement is marked by the black square in figure 9 at time = 10 min—and we have quantified the specific interfacial area between gas and the other phases (water, oil and solid) before and after its retraction from the narrower regions of the pore space. We notice, in figure 10*b*, that there is a large increase in the gas saturation caused by the three-phase Haines jump at time = 10 min—shown by the red square. Gas re-arranges itself in the pore space during the three-phase Haines jump, flowing towards regions of low gas pressure to enable the rapid filling, which causes it to retract from the high-pressure regions (throats), disconnecting the gas phase. This is shown in figure 10*c*, where gas has a lower specific interfacial area with the other phases of 6.7 mm^−1^ at time = 10 min after the three-phase Haines jump compared to time = 8.78 min, where gas had a specific interfacial area of 6.9 mm^−1^—gas-specific interfacial area is quantified in the region marked with the black dashed line.

**Figure 11.**
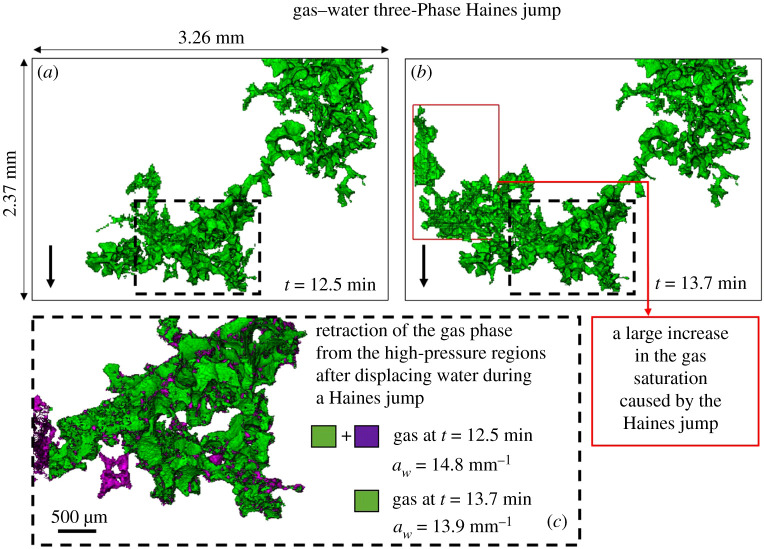
Three-dimensional images of the gas phase at different time steps illustrating the occurrence of a three-phase Haines jump during the displacement of water by gas in the oil-wet pore space. (*a*) and (*b*) show the difference in gas saturation before and after the three-phase Haines jump. (*c*) The specific interfacial area between gas and the rest of the phases (water, oil and solid) is lower in the high-pressure region, marked by the dashed line, after the three-phase Haines jump owing to gas retraction. The black arrow points towards the direction of flow. (Online version in colour.)

The occurrence of a three-phase Haines jump during the displacement of water by gas is shown in figure 11—this displacement event is shown by the black square in figure 9 at time = 13.7 min. Again, we observe that multiple pores were filled during the displacement, resulting in a large increase in the gas saturation at time = 13.7 min. Similarly, gas has retracted from the high-pressure regions reducing its specific interfacial area with the other phases—in the dashed box—from 14.8 mm^−1^ at time = 12.5 min to 13.9 mm^−1^ at time = 13.7 min. In this case, gas retraction is more pronounced during the gas–water Haines jump (0.9 mm^−1^) than during the gas–oil one (0.2 mm^−1^). Since neither gas nor water forms layers, all the displacements are piston-like. However, gas is wetting to water, and so the initial advance is an imbibition process.

*Water connectivity and trapping*. Water, the most non-wetting, can become locally disconnected in the pore space during GI. Since gas, intermediate-wet, does not form spreading layers because of its large and negative spreading coefficient (*C_sg_* = −22.8 mN m^−1^; table 1), water is principally trapped by oil, the most wetting phase, rather than gas. An illustration of this is shown in electronic supplementary material, figure S4, where the incoming gas front only displaces some of the resident water, disconnecting it from the main water body; the trapped water cluster is surrounded by both oil and gas. Nevertheless, we observe that, in general, there is a single connected cluster across the system that contains most of the water throughout GI (see electronic supplementary material, figure S5) since gas, although more wetting than water, cannot trap water by snap-off, which is the principal capillary trapping process, as it does not spread in layers [31]. This is different from the oil-wet micromodel and laboratory micro-tomography studies, where the injection of gas disconnected the water phase in the pore space [18,29]. However, in this previous work [15], the gas was near-miscible with the oil, and could form spreading layers to trap water by snap-off.

*Oil layers.* As mentioned in §2a(ii), the large and negative spreading coefficients of gas and water (*C_sg_* = −22.8 mN m^−1^ and *C_sw_* = −104.6 mN m^−1^; table 1) prevent them from forming spreading layers in the pore space. Oil not only spreads in layers sandwiched between gas and water, *C_so_* = 0.4 mN m^−1^ (table 1), but also exists in wetting layers close to the solid surface with gas or water occupying the centre of the pore space. Owing to the lack of spreading water and gas layers, oil is the only phase that is always hydraulically connected from the inlet to the outlet of the porous medium. This increases its connectivity in the pore space, allowing it to flow even at very low oil saturations. This is in contrast with water-wet systems, where both oil and water are connected in the pore space; water is hydraulically connected through wetting layers and oil through spreading layers [16].

Electronic supplementary material, figure S6 shows three-dimensional thickness maps of oil layers visualized at the end of WF and GI on a subset of size 1.4 × 1.4 × 1.4 mm^3^. The thickness was defined as the diameter of the largest sphere (maximal ball) that could fit entirely within the oil phase [83]. The average oil layer thicknesses after WF and GI were 17 µm and 14 µm, respectively. As one would expect, there were fewer and thinner oil layers in the pore space after GI owing to the efficient displacement of oil by gas and/or drainage of oil through wetting layers. This is different from observations made on a mixed-wet system, where more oil layers were observed after GI [49]: in these experiments, gas–oil–water double displacement allowed oil to push water out of the pore space, increasing the thickness of wetting and spreading layers and there was little direct displacement of water by gas. In our experiment, gas displaces water directly, as well as oil, removing both phases out of the pore space.

### (d) Minkowski functionals

**Figure 12.**
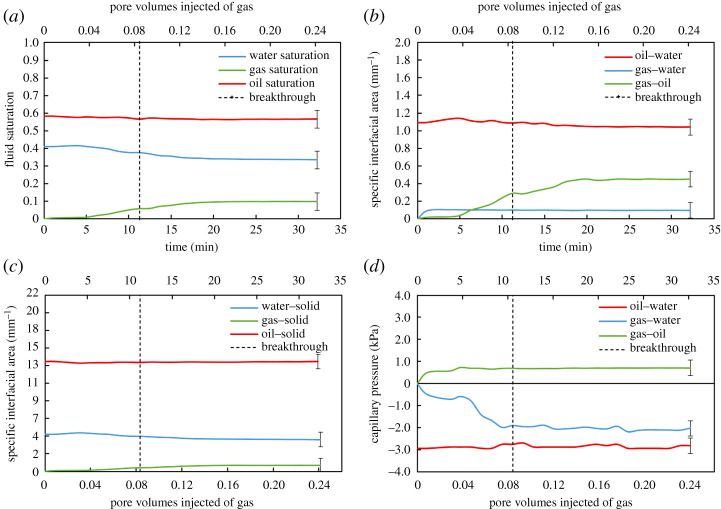
The evolution of Minkowski functionals—(*a*) saturation, (*b*) fluid–fluid specific interfacial area, (*c*) fluid–solid specific interfacial area and (*d*) capillary pressure—during gas injection in the dynamically imaged section of the oil-wet rock. The vertical dashed line represents the time of gas breakthrough in the imaged field of view. Error bars indicate uncertainty in the measurement. (Online version in colour.)

To obtain a complete characterization of the dynamics of three-phase flow, we quantified the evolution of the three-dimensional Minkowski functionals—saturations, interfacial areas and curvatures—during GI. In figure 12, measured on the dynamic images, we plot fluid saturations, fluid–fluid specific interfacial areas, fluid–solid specific interfacial areas and fluid–fluid capillary pressures against time and the corresponding PV of gas injected.

Note that the fluid saturations depicted in figure 12*a* are measured on the dynamically imaged section of the rock and are different from the saturations quantified on the whole sample in §3b. At the start of GI, the gas saturation increases very slowly until time = 5 min, where gas displaces oil and large amounts of water out of the dynamically imaged pore space. There is a slightly larger drop in water saturation compared to oil saturation during GI. However, it is important to note that the favoured displacement of water by gas over oil by gas is only seen in the dynamically imaged section, as saturation measurements on the whole sample (§3b) show that GI recovers 30 ± 5% of the resident oil, while only 16 ± 5% of water is produced. Moreover, we observe that the gas saturation further increases after gas breakthrough in the imaged section.

Figure 12*b*,*c* shows the evolution of fluid–fluid and fluid–solid specific interfacial areas, respectively, with time. At the beginning of GI, at low gas saturations, the interfacial area between gas and oil is very small. As the gas saturation increases, the gas–oil specific interfacial area rises linearly with time since oil is wetting to gas in the pore space; oil wetting and spreading layers surround gas in the centres of the intermediate-sized pores. However, the gas–oil specific interfacial area remains smaller than that between oil–water since the gas saturation is much lower than the water saturation. There is an abrupt increase in the gas–water interfacial area at the start of GI, which then remains constant throughout the displacement. This is attributed to spreading of oil layers sandwiched between gas and water, preventing their direct contact in the pore space. Furthermore, the low gas saturation results in a very low gas–solid interfacial area in the pore space. The oil–solid interfacial area is the highest owing to oil being the most wetting phase; oil resides in thick wetting layers next to the solid surface.

**Figure 13.**
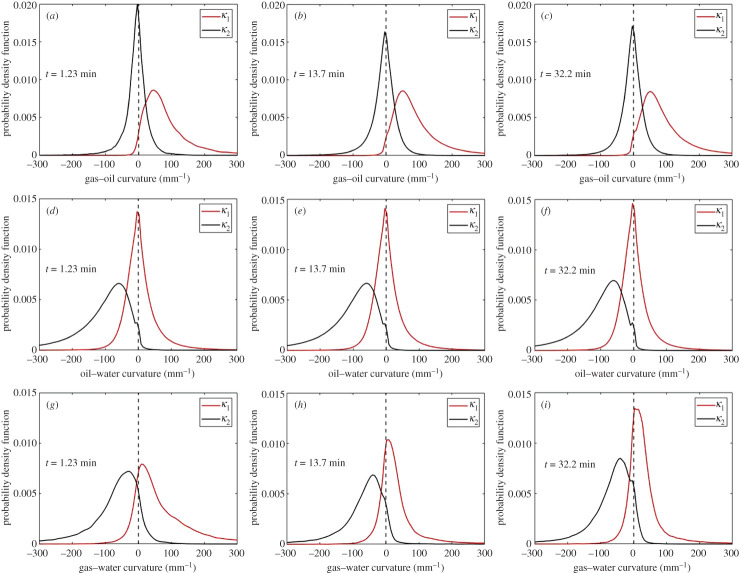
Probability distributions of the two principal curvatures, *κ*_1_ and *κ*_2_, at the (*a*–*c*) gas–oil interface, (*d*–*f*) oil–water interface and (*g*–*i*) gas–water interface plotted at different time steps during gas injection in the oil-wet porous medium. *κ*_1_ is defined to be the larger curvature. (Online version in colour.)

The two principal curvatures (*κ*_1_ and *κ*_2_) of the oil–water, gas–water and gas–oil interfaces were quantified during the displacement (figure 13). The sum of the two curvatures—the total curvature (*κ*)—was then calculated and substituted in the Young–Laplace equation (2.3), to obtain the fluid–fluid capillary pressures during GI. The results are shown in figure 12*d*. The oil–water capillary pressure remains approximately constant throughout the displacement with an average value of −3.0 kPa. A negative capillary pressure between oil and water indicates that the macro-pores are indeed oil-wet such that, on average, water bulges into oil with a higher pressure. The measured gas–water capillary pressure decreases during the displacement, reaching a value of −2.0 kPa at the end of GI. A negative gas–water capillary pressure indicates that gas is more wetting to the rock surface than water. This confirms the reported wettability order of oil–gas–water from most to least wetting in §3a. Moreover, once gas is injected, the capillary pressure between gas and oil reaches a threshold value, after which it remains constant during the displacement. The gas–oil capillary pressure is positive, since gas is less wetting than oil.

As mentioned previously, in section ‘Saturation, specific interfacial area and curvature’ , the two principal curvatures of the fluid–fluid interface can be used to study the connectedness of the fluid phases in the pore space. The fluid–fluid connectivity can be characterized by investigating the product of the principal curvatures (*κ*_1_ ×  *κ*_2_), also known as the Gaussian curvature [78]. A negative Gaussian curvature is indicative of well-connected phases in the pore space, while a positive value indicates that the two phases form trapped clusters.

Figure 13*a–c* shows that *κ*_1_ and *κ*_2_ of the gas–oil interface have opposite signs, resulting in a very negative Gaussian curvature between gas and oil during GI. This indicates that oil is well connected in the pore space when in contact with gas, in wetting and spreading layers. Similarly, *κ*_1_ and *κ*_2_ of the oil–water interface have opposite signs (figure 13*d–f*), indicating that oil and water are well connected, especially that water remains highly connected in the larger pores surrounded by oil layers during GI (see section ‘Oil layers’ and electronic supplementary material, figure S5).

Observations on the dynamic behaviour of *κ*_1_ and *κ*_2_ between gas and water are the most interesting (figure 13*g–i*). There are two important points to make. (i) It is evident from the distribution of *κ*_1_ and *κ*_2_ that the gas–water interface has a less negative Gaussian curvature than the gas–oil and oil–water interfaces, implying that gas and water are less connected in the pore space. This makes sense since neither water nor gas forms spreading layers because of their large and negative spreading coefficients (*C_sg_* = −22.8 mN m^−1^ and *C_sw_* = −104.6 mN m^−1^; table 1); the spreading of a fluid phase in layers enhances its connectivity in the pore space. Furthermore, as illustrated in figures 8 and 9, the gas is disconnected throughout GI. (ii) We observe that, as GI proceeds, *κ*_1_ gets smaller with time, consistent with there being more gas clusters as injection proceeds (figure 8).

## 4. Conclusion and future work

We investigated the pore-scale dynamics of three-phase flow in a hydrophobic porous medium. Synchrotron X-ray imaging, with high spatial and temporal resolutions (3.5 µm and 74 s), was used to visualize the displacement of fluids inside the pore space during immiscible GI in an oil-wet reservoir rock. Subsequent to altering the wettability of the rock surfaces, water was injected into the oil-saturated pore space, which was then followed by GI. The use of a synchrotron light source allowed us to characterize, *in situ*, wettability order, pore occupancy, fluid saturations, connectivity, direct and double displacement events, and Minkowski functionals, which provided insights into fluid–fluid connectivity and trapping.

Measurements of geometric and thermodynamic contact angles confirmed that the medium was oil-wet (hydrophobic), with oil–water contact angles greater than 90°. The characterization of geometric contact angles, measured locally, was insufficient to determine the wettability order in the system as it indicated that gas and water are neutrally wetting to the rock surface. In contrast, the estimation of thermodynamic contact angles, calculated during displacement using energy balance, demonstrated that the wettability order is oil–gas–water from most to least wetting. This was further supported by pore occupancy—where oil occupied the smallest pores, gas the intermediate pores and water the largest pores—and capillary pressure measurements, which displayed negative oil–water and gas–water pressures and a positive gas–oil pressure. Overall, this analysis showed that geometric contact angles, measured on static interfaces, tend to underestimate the contact angles encountered during displacement, but that using an energy balance can correctly capture a representative wettability in three-phase flow.

We imaged the fluid configurations during GI, which illustrated that gas invades the porous medium in the form of disconnected clusters; gas being the intermediate-wet phase is not connected in the pore space. When gas displaced either oil or water, it rapidly filled multiple pores, significantly increasing the gas saturation in the pore space. This rapid filling was accompanied by retraction of gas from some of the further regions, which disconnected the gas ganglia permanently in the pore space; the disconnected gas ganglia do not reconnect as GI continued. We call this phenomenon a three-phase Haines jump. Unlike in two-phase flow, the injected phase remained disconnected with displacement facilitated by double and multiple displacements. This dynamics is unique to three-phase flow, and is distinct from ganglion movement in two-phase flow, which only occurs under viscous-dominated flow conditions.

As gas invaded the pore space, it displaced oil and water in direct gas–water and gas–oil displacements, as well as double and multiple gas–oil–water displacement. No evidence of significant gas–water–oil double displacement was observed; as water is displaced by gas, water follows the easiest path to escape the pore space, which is its own path since it resides in the largest pores being the most non-wetting phase, and, therefore, does not displace oil. During GI, water maintains its connectivity through the larger pores, while oil remains hydraulically connected through wetting layers and spreading oil layers. Some water gets trapped in the porous medium during GI.

We quantified the Minkowski functionals—saturations, interfacial areas and curvatures—during GI to provide a complete description of the topology of fluids in the pore space, fluid–fluid connectivity and trapping. The oil–water specific interfacial area was the highest, while the gas–water area was the lowest owing to the spreading of oil in layers sandwiched between gas and water, and hence preventing their direct contact in the pore space. Quantification of the two principal curvatures of the oil–water, gas–water and gas–oil interfaces provided details on the connectivity of the phases. The results indicated that oil has a good connectivity with gas and water in the pore space. This was attributed to the oil-wet nature of the rock, since oil is confined in wetting layers close to the solid surface surrounding the gas and water phases in the centre of the pores. The analysis further confirmed the poor connectivity of the gas, which is broken up into discrete clusters as injection proceeds. This has significant implications for the design of safe gas storage, improved oil recovery, contaminant removal in soils and three-phase flow in microfluidic devices.

This work can be used to validate three-phase flow pore-scale network models and to develop three-phase flow numerical simulators. Experiments on additional samples could test the reproducibility of the results presented here. Future work should focus on quantifying the relative permeability of the phases in oil-wet rocks by measuring the pressure drop across the sample to confirm the low gas mobility anticipated as a result of gas advancing in disconnected clusters. Furthermore, future work can study the dynamics of three-phase flow at near-miscible gas–oil conditions in water-wet and oil-wet porous media. This will help to assess the impact of (i) the absence of oil layers in water-wet systems and (ii) the formation of gas spreading layers in oil-wet systems, on the pore-scale displacement events. The experimental and image analysis methodology presented in this work can be used to design the flow and trapping of three fluid phases in microfluidic devices, fuel cells, carbon storage and contaminant remediation in soils.

## Supplementary Material

Supplementary Material

## Supplementary Material

Movie S1 - Two-phase water Injection in an oil-wet system

## Supplementary Material

Movie S2 - Three-phase gas Injection in an oil-wet system

## Data accessibility

This paper contains confidential images of rocks extracted from a large hydrocarbon-producing reservoir in the Middle East; therefore, they cannot be shared on public platforms. However, sufficient experimental and numerical data have been provided in the paper to make the results reproducible by other scientists. Furthermore, access to these images might be granted by contacting the authors directly.
