# Supplementary material for: Three-phase flow displacement dynamics and Haines jumps in a hydrophobic porous medium
Source: Proc Math Phys Eng Sci. 2020 Dec 23;476(2244):20200671. doi: 10.1098/rspa.2020.0671 (PMC7776970; doi:10.1098/rspa.2020.0671)
Supplement: Supplementary Material [file rspa20200671supp1.docx]

#### Three-Phase Flow Displacement Dynamics in Hydrophobic Porous Media

Abdulla Alhosani^a^*, Alessio Scanziani^a^, Qingyang Lin^a^, Ahmed Selem^a^, Ziqing Pan^b^, Martin J. Blunt^a^, Branko Bijeljic^a^

*^a^Imperial College London, Department of Earth Science and Engineering, London, UK*

*^b^Imperial College London, Department of Chemical Engineering, London, UK*

* *Correspondence*: Abdulla.alhosani17@imperial.ac.uk

Table S1. Composition of the crude oil, extracted from a producing reservoir in the Middle East, used to alter the wettability of the reservoir rock. From [Alhammadi, et al. [1]](#_ENREF_1).

| Crude oil properties | | |
| --- | --- | --- |
| Density at 21 ^o^C | 830 ± 5 | kg/m^3^ |
| Saturates | 55.25 | wt% |
| Aromatics | 38.07 | wt% |
| Resins | 6.22 | wt% |
| Asphaltenes | 0.46 | wt% |
| Total Acid Number | 0.24 | mg KOH/g |
| Total Base Number | 356 | ppm |


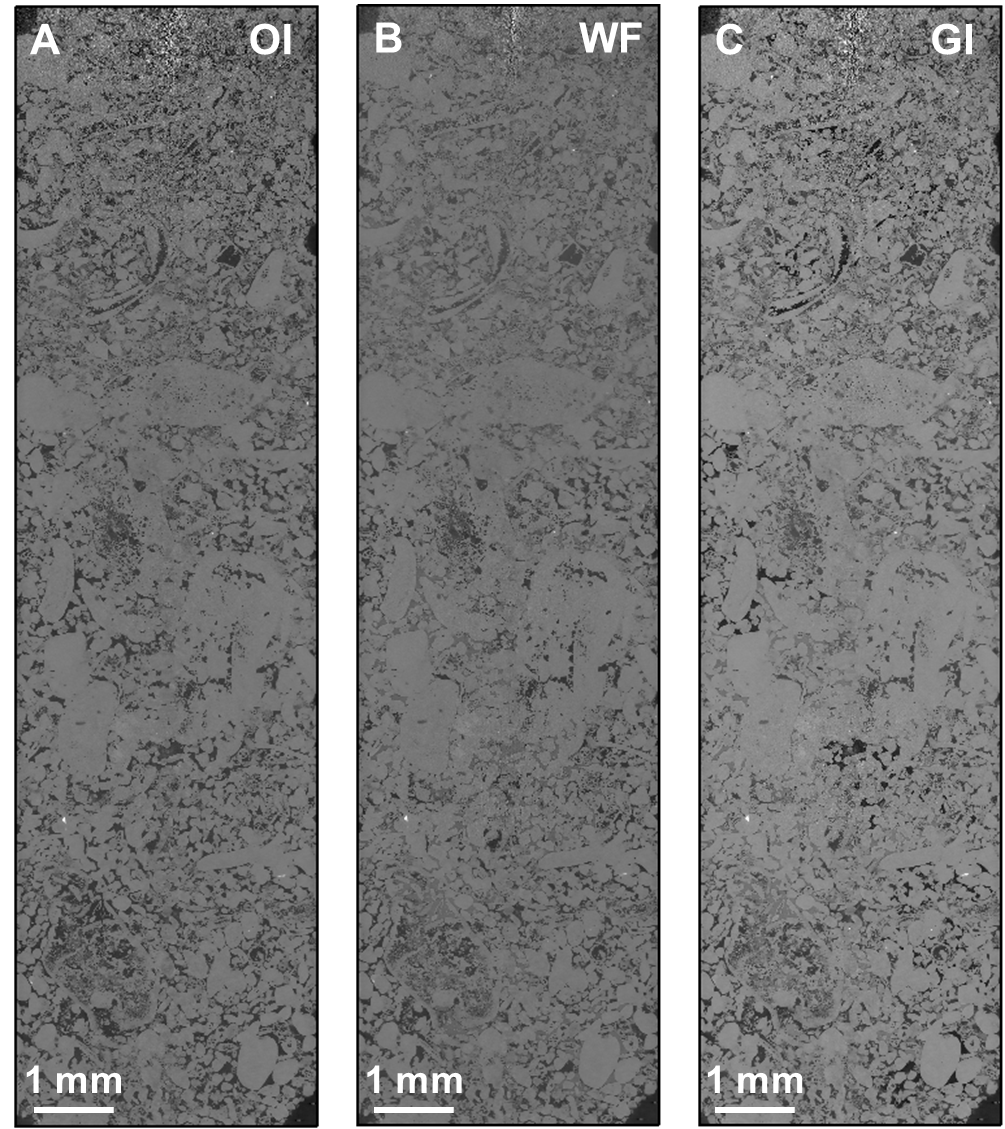


Figure S1. Raw static images with a 3.57 µm/voxel resolution of the whole sample after each injection sequence: (a) after oil injection [OI], (b) after water flooding [WF] and (c) after gas injection [GI]. In (a), rock is the light phase and oil is the dark phase. In (b) and (c), the order from darkest to brightest is: oil-water-rock and gas-oil-water-rock respectively.


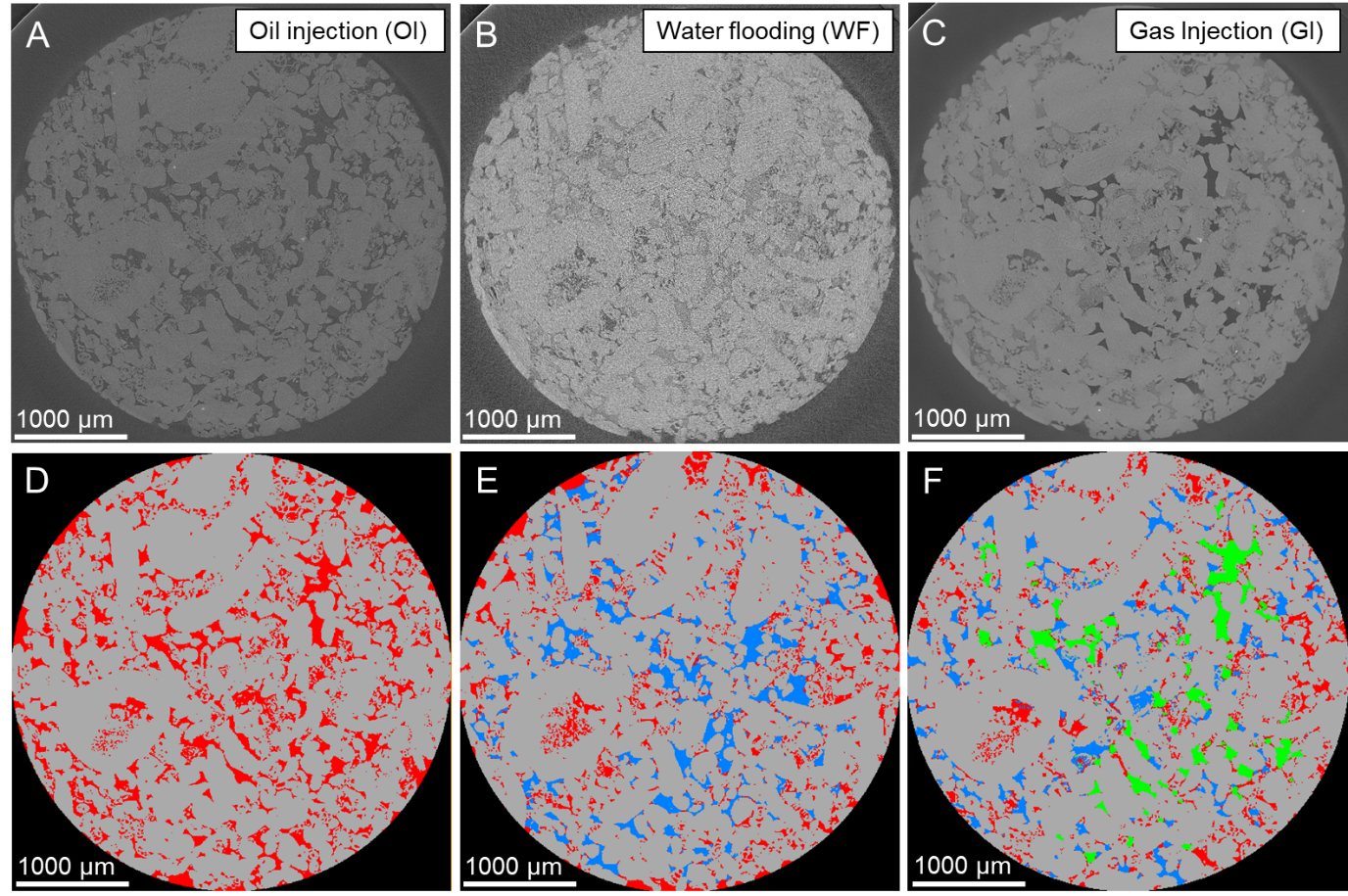


Figure S2. Image segmentation. (Top row) raw images of the sample with a 3.57 µm/voxel resolution after: (a) oil injection [OI], (b) water flooding [WF] and (c) gas injection [GI]. (Bottom row) Segmentation of the images in the top row using WEKA segmentation method with mean and variance texture filters. These images were selected to show the accuracy of segmentation for two, three and four phases. In (a), rock is the light phase and oil is the dark phase. In (b) and (c), the order from darkest to brightest is: oil-water-rock and gas-oil-water-rock respectively. In the segmented images, gas is shown in green, rock in grey, oil in red and water in blue.


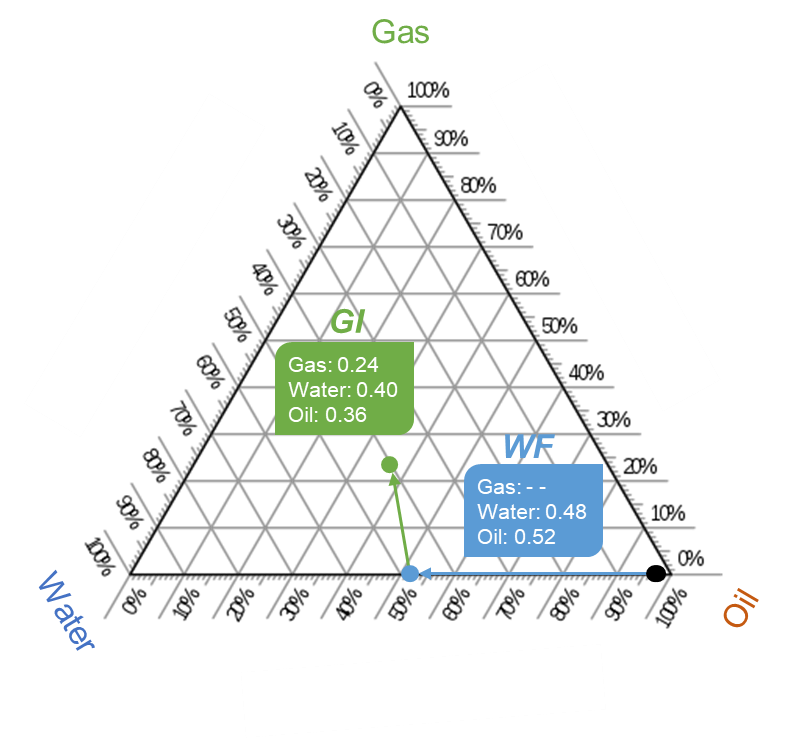


Figure S3. A ternary diagram showing the end-point saturations of oil, water and gas after water flooding [WF] and gas injection [GI]. Initially, the rock is almost fully saturated with oil in the macro pores (black point), then water is injected during WF resulting in the end saturations shown in the blue point, followed by gas injection during GI (green point).


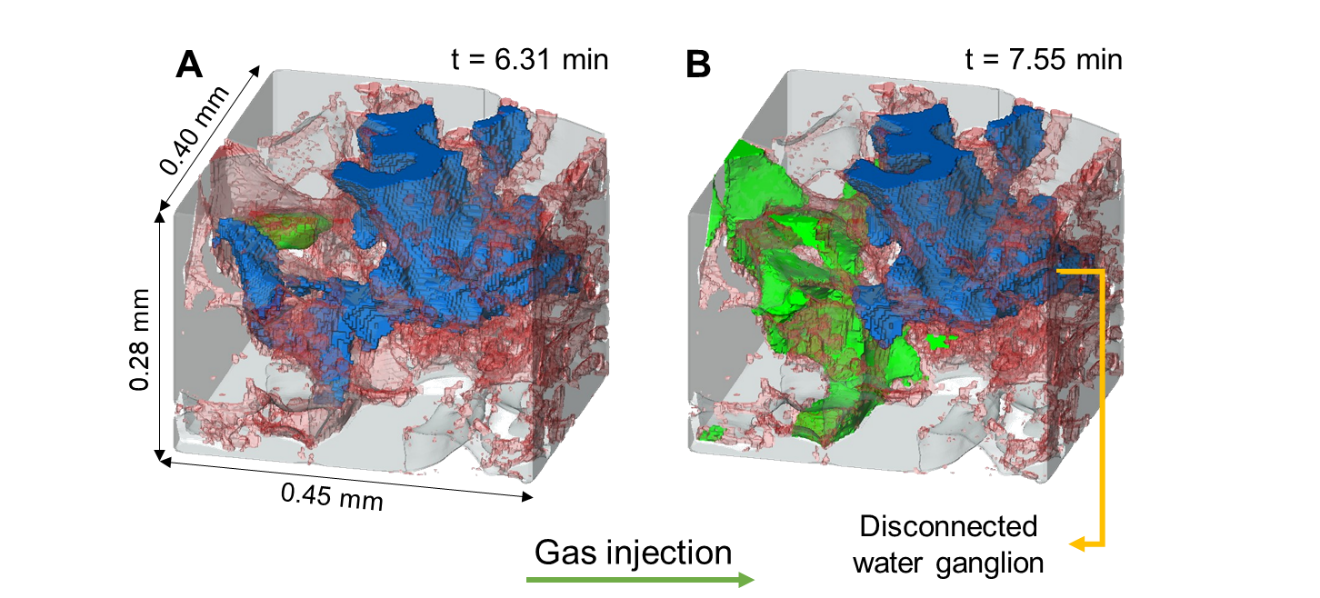


Figure S4. Three-dimensional images of a small section of the pore space showing the trapping of water during gas injection in the oil-wet rock. Oil is shown in red, water in blue, gas in green while the rock is rendered transparent to permit the visualization of fluid configurations in the pore space.


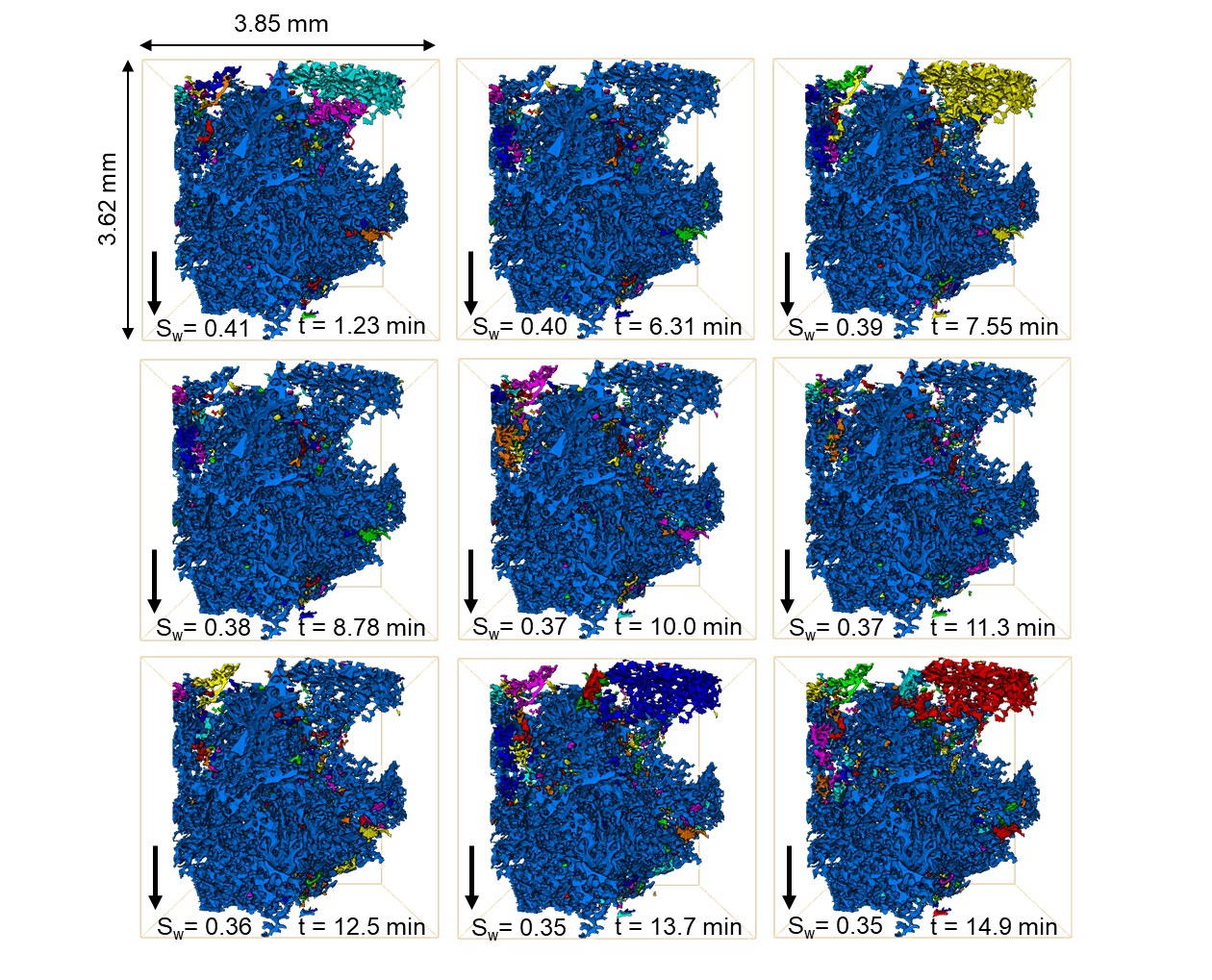


Figure S5. Three-dimensional maps of the water connectivity in the pore space during GI shown at different time-steps. Each disconnected water cluster is labeled with a different color. The black arrow points towards the direction of flow. *S_w_* is the gas saturation in the imaged section, while *t* is time.


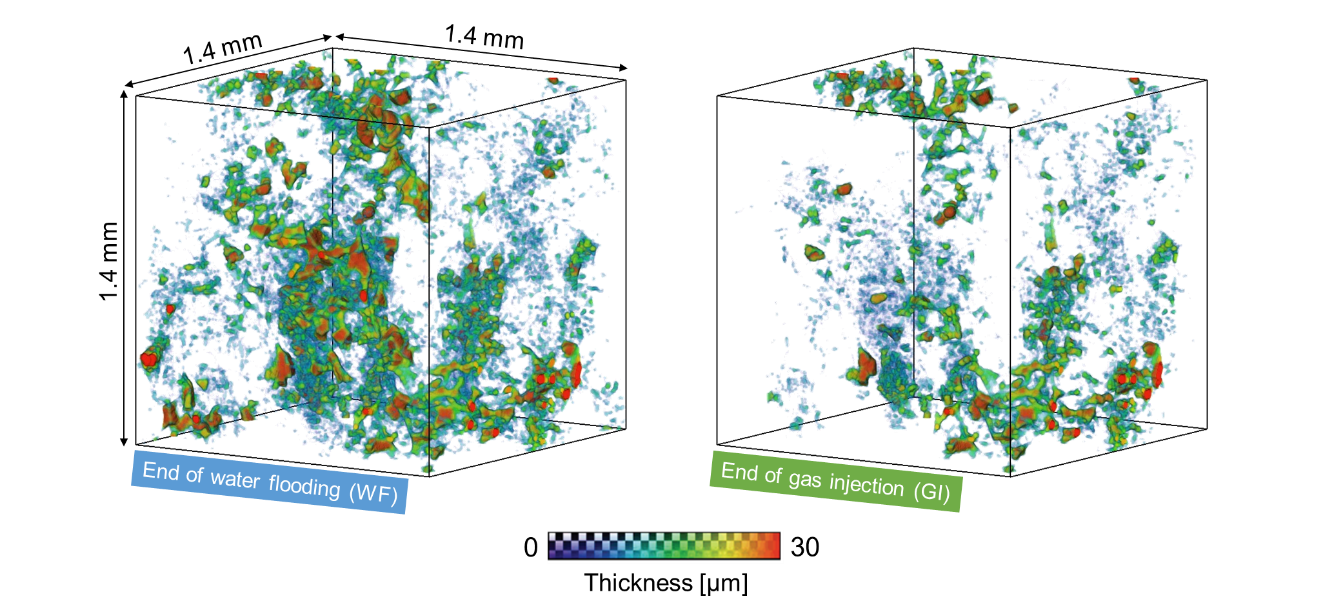


Figure S6. Three-dimensional thickness maps of oil layers shown at the end of (left) water flooding [WF] and (right) gas injection [GI]. The oil phase was isolated and maximal balls were fitted to its structure to obtain the thickness maps.

Three-phase thermodynamic contact angle: data from segmented images

| Time [s] | S_w_ | S_g_ | a_ow_ [mm^-1^] | a_gw_ [mm^-1^] | a_go_ [mm^-1^] | a_ws_ [mm^-1^] | a_gs_ [mm^-1^] | κ _ow_ [mm^-1^] | κ _go_ [mm^-1^] |
| --- | --- | --- | --- | --- | --- | --- | --- | --- | --- |
| 1.2333333 | 0.41112 | 0.0059708 | 1.094409938 | 0.096543823 | 0.019870807 | 4.625258799 | 0.089184265 | -81.71428571 | 45.41714286 |
| 3.85 | 0.41581 | 0.0080891 | 1.134023464 | 0.104833678 | 0.024655625 | 4.788405797 | 0.108662526 | -81.71428571 | 50.98285714 |
| 5.0833333 | 0.41076 | 0.0101 | 1.14084196 | 0.104400276 | 0.041289165 | 4.746445825 | 0.128146308 | -78.8 | 65.31428571 |
| 6.3166667 | 0.40444 | 0.019939 | 1.112574189 | 0.102539683 | 0.099254658 | 4.661973775 | 0.193195307 | -79.65714286 | 62.22857143 |
| 7.55 | 0.39758 | 0.027043 | 1.100096618 | 0.103765355 | 0.132938578 | 4.605383023 | 0.250009662 | -80.22857143 | 62 |
| 8.7833333 | 0.38331 | 0.039855 | 1.115638371 | 0.102777088 | 0.171641132 | 4.459627329 | 0.336645963 | -80.45714286 | 58.4 |
| 10.016667 | 0.37657 | 0.050954 | 1.09294686 | 0.100436163 | 0.238694272 | 4.382332643 | 0.419296066 | -81.31428571 | 61.77142857 |
| 11.25 | 0.37634 | 0.057936 | 1.086625259 | 0.101741891 | 0.290545204 | 4.376535542 | 0.45915804 | -81.2 | 62.11428571 |
| 12.483333 | 0.36884 | 0.059263 | 1.094271912 | 0.099922705 | 0.283395445 | 4.305590062 | 0.472574189 | -79.02857143 | 60.68571429 |
| 13.716667 | 0.35851 | 0.071233 | 1.076935818 | 0.100132505 | 0.31094548 | 4.251207729 | 0.549868875 | -81.2 | 60.68571429 |
| 14.95 | 0.35608 | 0.076298 | 1.083395445 | 0.101013112 | 0.335983437 | 4.223878537 | 0.585866115 | -80.8 | 61.25714286 |
| 16.183333 | 0.34824 | 0.084622 | 1.057612146 | 0.099743271 | 0.36389234 | 4.132229124 | 0.650655625 | -81.48571429 | 60.97142857 |
| 17.416667 | 0.34464 | 0.08947 | 1.059820566 | 0.098824017 | 0.417860594 | 4.091373361 | 0.684030366 | -81.65714286 | 62.34285714 |
| 18.65 | 0.34344 | 0.0934 | 1.052891649 | 0.096300897 | 0.438178054 | 4.072325742 | 0.718067633 | -81.42857143 | 62.85714286 |
| 19.883333 | 0.34029 | 0.095393 | 1.047011732 | 0.099986197 | 0.451704624 | 4.053830228 | 0.739627329 | -81.42857143 | 62.22857143 |
| 21.116667 | 0.34015 | 0.096611 | 1.044223602 | 0.099204969 | 0.436521739 | 4.049137336 | 0.748240166 | -81.65714286 | 63.02857143 |
| 22.35 | 0.33944 | 0.097581 | 1.045327812 | 0.099064182 | 0.440138026 | 4.038647343 | 0.759337474 | -81.48571429 | 62.68571429 |
| 23.583333 | 0.33785 | 0.097655 | 1.040717736 | 0.097416149 | 0.451290545 | 4.029537612 | 0.748985507 | -81.42857143 | 63.25714286 |
| 24.816667 | 0.3374 | 0.098019 | 1.043119393 | 0.097783299 | 0.449744651 | 4.010766046 | 0.748295376 | -82.05714286 | 63.14285714 |
| 26.05 | 0.33741 | 0.09758 | 1.045548654 | 0.098271912 | 0.446321601 | 4.010489993 | 0.74252588 | -82.05714286 | 63.08571429 |
| 27.283333 | 0.33715 | 0.098558 | 1.04173913 | 0.099149758 | 0.452505176 | 4.006073154 | 0.756549344 | -82 | 63.25714286 |
| 28.516667 | 0.33675 | 0.098128 | 1.043146998 | 0.09794617 | 0.448833678 | 3.994478951 | 0.750089717 | -81.88571429 | 63.65714286 |
| 29.75 | 0.33675 | 0.098551 | 1.039392685 | 0.099514148 | 0.447867495 | 3.998067633 | 0.758095238 | -82 | 63.31428571 |
| 30.983333 | 0.33629 | 0.097977 | 1.038426501 | 0.098570048 | 0.44621118 | 3.98426501 | 0.748378192 | -81.54285714 | 63.42857143 |
| 32.216667 | 0.3355 | 0.098218 | 1.039889579 | 0.097659075 | 0.451511387 | 3.965217391 | 0.750117322 | -81.88571429 | 62.97142857 |

Three-phase thermodynamic contact angle: differences

| Time [s] | ΔS_w_ | ΔS_g_ | Δa_ow_ [mm^-1^] | Δa_gw_ [mm^-1^] | Δa_go_ [mm^-1^] | Δa_ws_ [mm^-1^] | Δa_gs_ [mm^-1^] | Δκ _ow_ [mm^-1^] | Δκ _go_ [mm^-1^] |
| --- | --- | --- | --- | --- | --- | --- | --- | --- | --- |
| 1.2333333 |  |  |  |  |  |  |  |  |  |
| 3.85 | 0.00469 | 0.002118 | 0.039614 | 0.00829 | 0.004785 | 0.163147 | 0.019478 | -81.7143 | 48.2 |
| 5.0833333 | -0.00505 | 0.002011 | 0.006818 | -0.00043 | 0.016634 | -0.04196 | 0.019484 | -80.2571 | 58.14857 |
| 6.3166667 | -0.00632 | 0.009839 | -0.02827 | -0.00186 | 0.057965 | -0.08447 | 0.065049 | -79.2286 | 63.77143 |
| 7.55 | -0.00686 | 0.007104 | -0.01248 | 0.001226 | 0.033684 | -0.05659 | 0.056814 | -79.9429 | 62.11429 |
| 8.7833333 | -0.01427 | 0.012812 | 0.015542 | -0.00099 | 0.038703 | -0.14576 | 0.086636 | -80.3429 | 60.2 |
| 10.016667 | -0.00674 | 0.011099 | -0.02269 | -0.00234 | 0.067053 | -0.07729 | 0.08265 | -80.8857 | 60.08571 |
| 11.25 | -0.00023 | 0.006982 | -0.00632 | 0.001306 | 0.051851 | -0.0058 | 0.039862 | -81.2571 | 61.94286 |
| 12.483333 | -0.0075 | 0.001327 | 0.007647 | -0.00182 | -0.00715 | -0.07095 | 0.013416 | -80.1143 | 61.4 |
| 13.716667 | -0.01033 | 0.01197 | -0.01734 | 0.00021 | 0.02755 | -0.05438 | 0.077295 | -80.1143 | 60.68571 |
| 14.95 | -0.00243 | 0.005065 | 0.00646 | 0.000881 | 0.025038 | -0.02733 | 0.035997 | -81 | 60.97143 |
| 16.183333 | -0.00784 | 0.008324 | -0.02578 | -0.00127 | 0.027909 | -0.09165 | 0.06479 | -81.1429 | 61.11429 |
| 17.416667 | -0.0036 | 0.004848 | 0.002208 | -0.00092 | 0.053968 | -0.04086 | 0.033375 | -81.5714 | 61.65714 |
| 18.65 | -0.0012 | 0.00393 | -0.00693 | -0.00252 | 0.020317 | -0.01905 | 0.034037 | -81.5429 | 62.6 |
| 19.883333 | -0.00315 | 0.001993 | -0.00588 | 0.003685 | 0.013527 | -0.0185 | 0.02156 | -81.4286 | 62.54286 |
| 21.116667 | -0.00014 | 0.001218 | -0.00279 | -0.00078 | -0.01518 | -0.00469 | 0.008613 | -81.5429 | 62.62857 |
| 22.35 | -0.00071 | 0.00097 | 0.001104 | -0.00014 | 0.003616 | -0.01049 | 0.011097 | -81.5714 | 62.85714 |
| 23.583333 | -0.00159 | 7.4E-05 | -0.00461 | -0.00165 | 0.011153 | -0.00911 | -0.01035 | -81.4571 | 62.97143 |
| 24.816667 | -0.00045 | 0.000364 | 0.002402 | 0.000367 | -0.00155 | -0.01877 | -0.00069 | -81.7429 | 63.2 |
| 26.05 | 1E-05 | -0.00044 | 0.002429 | 0.000489 | -0.00342 | -0.00028 | -0.00577 | -82.0571 | 63.11429 |
| 27.283333 | -0.00026 | 0.000978 | -0.00381 | 0.000878 | 0.006184 | -0.00442 | 0.014023 | -82.0286 | 63.17143 |
| 28.516667 | -0.0004 | -0.00043 | 0.001408 | -0.0012 | -0.00367 | -0.01159 | -0.00646 | -81.9429 | 63.45714 |
| 29.75 | 0 | 0.000423 | -0.00375 | 0.001568 | -0.00097 | 0.003589 | 0.008006 | -81.9429 | 63.48571 |
| 30.983333 | -0.00046 | -0.00057 | -0.00097 | -0.00094 | -0.00166 | -0.0138 | -0.00972 | -81.7714 | 63.37143 |
| 32.216667 | -0.00079 | 0.000241 | 0.001463 | -0.00091 | 0.0053 | -0.01905 | 0.001739 | -81.7143 | 63.2 |

Three-phase thermodynamic contact angle: equation solver

| φ | σ_ow_ [mN/m] | σ_gw_ [mN/m] | σ_go_ [mN/m] |
| --- | --- | --- | --- |
| 0.123889 | 52.1 | 63.7 | 11.2 |

Equation:

$$\left( \Delta a_{gs}\cos\theta_{go}+\Delta a_{go}- \kappa_{go}\phi\Delta S_{g} \right)\sigma_{go}+\Delta a_{gw}\sigma_{gw}-\left( \Delta a_{ws}\cos\theta_{ow}+\Delta a_{ow}- \kappa_{ow}\phi\Delta S_{w} \right)\sigma_{ow}=0$$

| Time [s] | θ_ow_ [^o^] | θ_go_ [^o^] | θ_gw_ [^o^] | Equation | Equation squared |  |  |
| --- | --- | --- | --- | --- | --- | --- | --- |
| 1.2333333 | 125.9768329 | 73 | 115.4087976 | 5.09 | 25.88093523451830000 |  |  |
| 3.85 | 125.9768329 | 73 | 115.4087976 | 1.75 | 3.05298877984986000 |  |  |
| 5.0833333 | 125.9768329 | 73 | 115.4087976 | -0.95 | 0.90836656155230100 |  |  |
| 6.3166667 | 125.9768329 | 73 | 115.4087976 | 1.19 | 1.40834661377316000 |  |  |
| 7.55 | 125.9768329 | 73 | 115.4087976 | 3.33 | 11.10774383811860000 |  |  |
| 8.7833333 | 125.9768329 | 73 | 115.4087976 | -0.08 | 0.00671078735413993 |  |  |
| 10.016667 | 125.9768329 | 73 | 115.4087976 | -0.19 | 0.03679307468710450 |  |  |
| 11.25 | 125.9768329 | 73 | 115.4087976 | 1.84 | 3.38638496442581000 |  |  |
| 12.483333 | 125.9768329 | 73 | 115.4087976 | 2.34 | 5.48097016963061000 |  |  |
| 13.716667 | 125.9768329 | 73 | 115.4087976 | 0.80 | 0.63432533576156900 |  |  |
| 14.95 | 125.9768329 | 73 | 115.4087976 | -0.30 | 0.09256067287663450 |  |  |
| 16.183333 | 125.9768329 | 73 | 115.4087976 | 1.00 | 1.00092845126223000 |  |  |
| 17.416667 | 125.9768329 | 73 | 115.4087976 | -0.48 | 0.22606144946790200 |  |  |
| 18.65 | 125.9768329 | 73 | 115.4087976 | 1.07 | 1.13864927673410000 |  |  |
| 19.883333 | 125.9768329 | 73 | 115.4087976 | -0.51 | 0.26282364794079400 |  |  |
| 21.116667 | 125.9768329 | 73 | 115.4087976 | 0.09 | 0.00875389020084656 |  |  |
| 22.35 | 125.9768329 | 73 | 115.4087976 | 0.30 | 0.08793812863086740 |  |  |
| 23.583333 | 125.9768329 | 73 | 115.4087976 | -0.24 | 0.05764097266186590 |  |  |
| 24.816667 | 125.9768329 | 73 | 115.4087976 | 0.13 | 0.01566457594811580 |  |  |
| 26.05 | 125.9768329 | 73 | 115.4087976 | -0.11 | 0.01223900821404890 |  |  |
| 27.283333 | 125.9768329 | 73 | 115.4087976 | -0.17 | 0.02924958605362340 |  |  |
| 28.516667 | 125.9768329 | 73 | 115.4087976 | -0.01 | 0.00006007891454337 |  |  |
| 29.75 | 125.9768329 | 73 | 115.4087976 | -0.29 | 0.08412078332074550 |  |  |
| 30.983333 | 125.9768329 | 73 | 115.4087976 | -0.10 | 0.01085526366843400 | Sum of squares | Solver |
| 32.216667 | 125.9768329 | 73 | 115.4087976 | 5.09 | 25.88093523451830000 | 54.9 | Change θ_wo_ and θ_og_ to minimize the sum |

References

[1] A. M. Alhammadi, A. AlRatrout, K. Singh, B. Bijeljic, and M. J. Blunt, "In situ characterization of mixed-wettability in a reservoir rock at subsurface conditions," *Scientific Reports,* vol. 7, no. 1, p. 10753, 2017/09/07 2017, doi: 10.1038/s41598-017-10992-w.
